# Resonance characteristics of tsunami in bay of Japan by the Hunga Tonga-Hunga Ha’apai volcano eruption on 15th January 2022

**DOI:** 10.1038/s41598-023-45601-6

**Published:** 2023-10-26

**Authors:** Kwanchai Pakoksung, Anawat Suppasri, Fumihiko Imamura

**Affiliations:** International Research Institute of Disaster Science, Tohoku University, Sendai, 980-8572 Japan

**Keywords:** Environmental sciences, Civil engineering, Physical oceanography, Natural hazards, Applied mathematics

## Abstract

The massive eruption of the Hunga Tonga-Hunga Ha’apai (HTHH) volcano in Tonga on 15 January 2022 at 04:15 UTC had a global impact and triggered an atmospheric wave and a tsunami. We first analyzed observation data from meteorological stations and tide gauges at 12 locations. Low-frequency trends in the observation data were removed by using a high-pass filter. Fourier and wavelet spectral analyses were applied to determine the frequency characteristics of the filtered data. Modal analysis was developed and used to investigate natural oscillation periods based on bathymetry. The results showed that the Lamb wave generated by the atmospheric pressure wave arrived ~ 7 and ~ 44 h after the eruption. The tsunami arrived ~ 11 and ~ 45 h after the eruption, which corresponded to the arrival time of the Lamb wave. The dominant periods of the Lamb waves were ~ 7.7 and ~ 7.5 min, and for the tsunamis they were ~ 9.9 and ~ 28.7 min. The periods derived from the spectral analysis matched the natural oscillation of the eigenperiod derived from the modal analysis, in eight out of the twelve stations. This study provides valuable insight and information regarding the nonseismic and far-field effects of tsunamis generated by volcanic eruptions.

## Introduction

The 2022 HTHH eruption produced an atmospheric pressure wave similar to a Lamb wave, which is characterized by a pressure pulse. Lamb waves transfer energy over a long distance to generate tsunami waves^1^. The nondispersive Lamb wave propagated along the horizontal direction at a speed of approximately 315 m/s^2,3^. The tsunami generated by the Lamb wave was induced by the massive volcanic eruption, which was similar to the tsunami that occurred following the 1883 Krakatau eruption^4^. Similarly, the tsunami generated by meteorological pressure in Lake Michigan in 1954 was also reported and modeled^5^.

**Figure 1:**
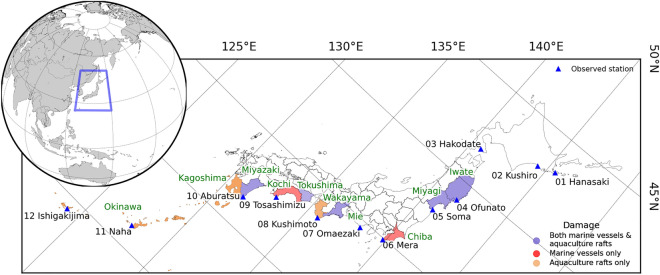
Study area. Observed station locations (atmospheric pressure and sea level) are presented by a blue triangle. These are arranged from north to south along the eastern coastline of Japan. The reported damage on the prefecture scale was obtained from Imamura et al.^12^ The figure was generated using Python version 3.8^66^ with the Matplotlib library^67^.

The tsunami was produced by the atmospheric pressure wave released from the 2022 HTHH eruption and propagated across the Pacific Ocean^6,7^. The maximum measured tsunami height was approximately 2.5 m in Central America^8^ and higher than 1 m in Chile, New Caledonia, Vanuatu, USA, and Japan^9–14^. The tsunami caused damage along the coastal area of the Pacific Ocean^12,15–18^. In Japan, the damage caused by this tsunami was located along the coastal areas on the eastern side of Japan and extended from the north to the south but was concentrated in the bays and ports. Damage was reported on marine vessels and aquaculture rafts^12^, as shown in Fig. 1, which indicates the location of damage at the prefecture scale. At present, the cause of the damage remains unclear, and may be solely related to the stiffness of the structure or perhaps to the physical tsunami mechanisms, such as resonance.

Tsunami resonance occurs when the tsunami wave period is similar to the natural oscillation of the water surface^19^ and is produced by the reflection and interference of tsunami waves at the edge of a bay or port. The amplification of the natural oscillation of the bay can generate a higher water level in the bay and increase run-up along the coast^19–22^. The energy generated by natural high-frequency tsunami oscillations can generate strong currents inside bays that may lead to damage on boats, ships, port infrastructure, and aquaculture rafts^23^. Therefore, understanding resonance characteristics is important to help identify tsunami hazards for coastal planning and emergency management^24–26^. Some previous studies have been conducted to investigate tsunami resonance around the Pacific Ocean. Cortés et al.^27^ calculated the longwave resonance to investigate the regions that had an increased potential for tsunamis, specifically within the northern coastal area of Chile. Abe^28^ investigated the dominant period of tsunami resonance in the bays of northern Japan and compared them with the spectra of tsunamis from the near-field and trans-Pacific location. Wang et al.^19^ investigated the tsunami dominant period from several trans-Pacific tsunami events in the Japan, Hokkaido, and Sanriku regions and compared them with the natural dominant period.

In this study, we first investigated the resonance characteristics of the 2022 HTHH trans-Pacific tsunami event in Japan. The investigation of resonance characteristics is based on the observations of atmospheric pressure and tsunami height in the bay along the eastern coast area of Japan (see Fig. 1) made by 12 stations, and some stations were in the areas damaged by the 2022 HTHH event. First, the collected data were quality controlled and processed by removing a low-frequency trend with a high-pass filter. We analyzed the filtered atmospheric pressure and tsunami height data from the 12 stations to investigate the potential relationship between the atmospheric pressure wave and tsunami wave. The frequency characteristics (dominant period) of atmospheric pressure and tsunami waves were calculated using both wavelet and Fourier spectral analyses. We also calculated the eigenmodes of the natural oscillation of the bay/port where the 12 stations were located to compare the dominant period from the HTHH 2022 event and the natural oscillation (on regional and local domains). This is the first study to investigate the resonance characteristics from a real a nonseismic tsunami event in Japan in which the tsunami was related to the atmospheric pressure waves generated by a volcanic eruption. We also provide theoretical information on the tsunami generated by the 2022 HTHH volcanic eruption.

## Results

### Data processing results

Atmospheric pressure data were collected from Weathernews Inc.^29^. We selected 12 meteorological stations along the eastern coast area of Japan that were located in relation to the selected tsunami gauge station (see Fig. 1), which had at a sampling rate of 1 min. The atmospheric pressure data were selected from January 14, 2022, at 00:00 UTC to January 19, 2022, at 00:00 UTC, which covers more 90 h following the HTHH eruption, as shown in Fig. S1a. The raw data were quality controlled to remove spikes, gaps, or redundant values. Next, a high-pass filter was applied to remove diurnal pressure oscillations^30^ with a cutoff of 6.67 × 10^–4^ Hz (1500 s), and the low-frequency trend is shown in Fig. S1a. The raw data subtracted from the low-frequency data become the filtered dataset (see Fig. S1b), which is summarized in Fig. S2. Tsunami height data were collected from 12 tide gauges along the eastern Japan coast (see Fig. 1). All gauge stations are in bays or ports, and their sampling rate is 1 min. Data from the 11 stations were obtained from the Intergovernmental Oceanographic Commission^31^: Hansaki, Kushiro, Hakodate, Ofunato, Mera, Omaezaki, Kushimoto, Tosashimizu, Aburatsu, Naha, and Ishigakijima. However, data from 1 station was obtained from the Geospatial Information Authority of Japan^32^, Soma. The selected time series of tsunami data covered the time between January 14, 2022, 00:00 UTC, and January 19, 2022, 00:00 UTC (see Fig. S3a). After the raw data were subjected to basic quality control, a high-pass filter was applied to remove the tidal signals with a cutoff of 6.25 × 10^−3^ Hz (160 s)^33–35^, and the low-frequency trend is shown in Fig. S3a. The dataset was filtered by calculating the differences between the raw data and the low-frequency data (see Fig. S3b) and are summarized in Fig. S4.

### Waveform analysis results

**Figure 2:**
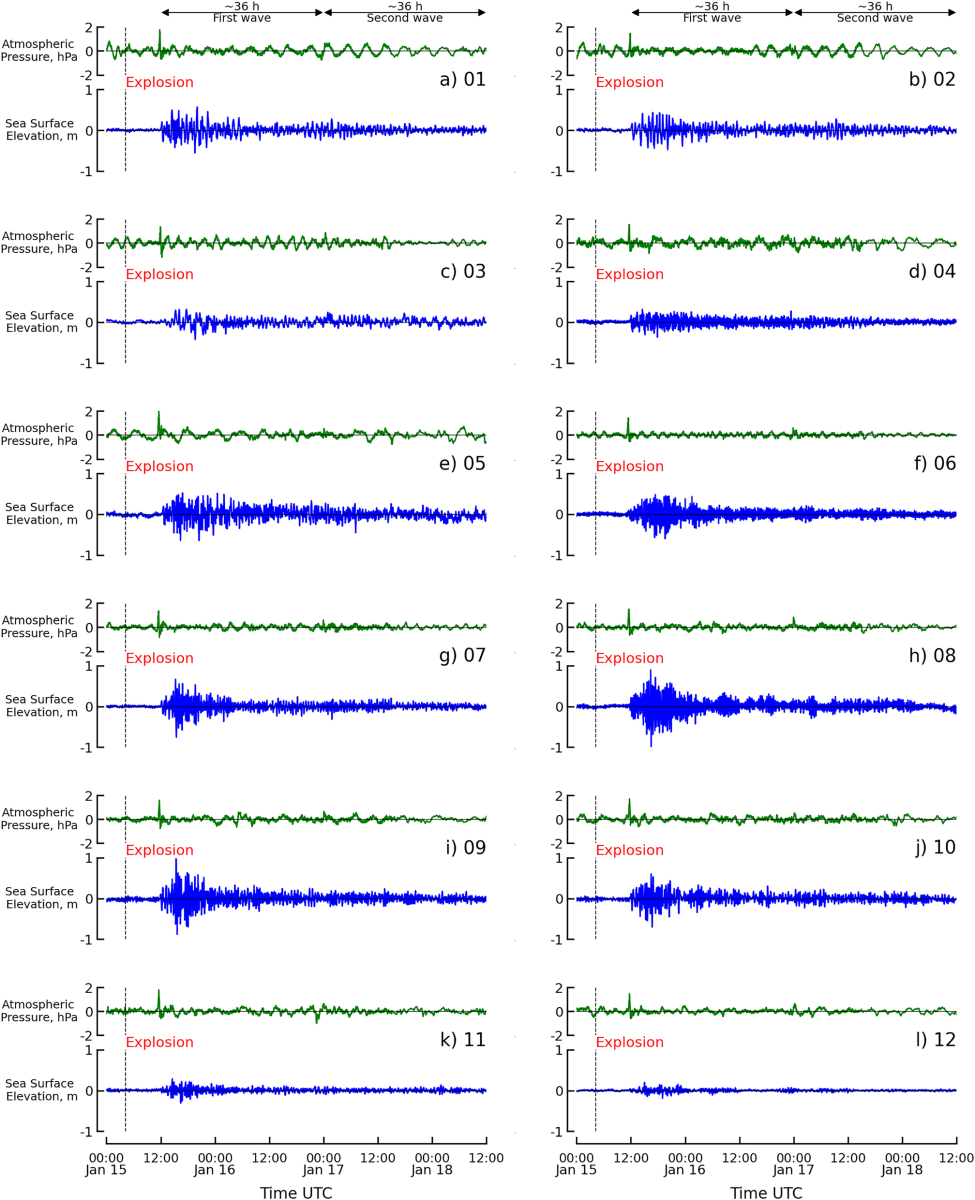
Time series of atmospheric pressure (green line) and sea level (blue line) records. The eruption of the HTHH volcano on 04:14:45 UTC, 15 January 2022, is indicated by the dashed black line. The first (12:00 UTC, 15 January 2022 to 00:00 UTC, 17 January 2022) and second (00:00 UTC, 17 January 2022 to 12:00 UTC, 18 January 2022) wave components were observed over approximately 36 h.

Atmospheric pressure and tsunami height from the filtered low-frequency data derived from the 12 stations are presented in Fig. 2. From the atmospheric pressure, two persistent waves (Lamb waves) were visible, with intervals of approximately 36 h. The duration of the first wave was from January 15, 2022, 11:00 UTC to January 17, 2022, 00:00 UTC, and the second wave occurred from January 17, 2022, 00:00 UTC to January 18, 2022, 12:00 UTC. The tsunami waves were also classified to be consistent with the duration of the Lamb wave, and two tsunami waves also arrived after the Lamb wave. The arrival times of both the Lamb wave and tsunami wave were generally consistent.

The data from the 12 stations indicate that the first Lamb wave arrived in Japan at approximately 11:00 (UTC) on 15 January 2022, approximately 7 h after the HTHH eruption, as shown in Fig. 2. The amplitude at the crest of the first Lamb wave was approximately 2 hPa with a wavelength of approximately 30 min. The shape of the first Lamb wave was represented by an N-wave shape and a small wave of approximately 0.5 hPa was visible following the crest.

The sea surface gradually varied and rose for approximately 2 h after the arrival of the first Lamb, as indicated by the tide gauge observations of the first tsunami wave. The height of the leading tsunami wave varied positively from 5 to 10 cm in the early phase and was apparent after 13:00 (UTC) on 15 January 2022. The maximum amplitude of the tsunami wave overall occurred in the later phase, as shown in Fig. 2. Hanasaki station, located at the north end of Hokkaido Island, reveals that the maximum height of the first tsunami was approximately 0.5 m and occurred at 15:30 UTC on 15 January 2022 (00:30 JST on 16 January 2022). Several peaks were continually generated over a period of 7 h until the largest peak of approximately 0.6 m occurred at 20:00 UTC on 15 January 2022 (5:00 JST on 16 January 2022). The Kushiro and Hakodate stations had a similar pattern to the Hanasaki station, with the first maximum peaks at also have heights approximately equal to 0.5 m and 0.3 m, respectively. Ofunato, Soma, and Mera stations, located on the eastern side of Japan, showed that the small first peak occurred at 15:00 UTC on 15 January 2022 (00:00 JST on 16 January 2022). Several peaks were continually generated until the largest peak was observed at 18:00 UTC on 15 January 2022 (03:00 JST on 16 January 2022). The largest tsunami peaks at Ofunato, Soma, and Mera were 0.25 m, 0.55 m, and 0.5 m, respectively. The stations on the western side of Japan are Omaezaki, Kushimoto, Tosashimizu, and Aburatsu, which revealed that the peaks on this coast were shorter in duration when compared to the peaks observed by the station in the northern area. The four stations recorded the largest peak at 15:00 UTC on 15 January 2022 (00:00 JST on 16 January 2022). The largest peaks at Omaesaki, Kushimoto, Tosashimizu, and Aburatsu were 0.75 m, 0.9 m, 1.0 m, and 0.55 m, respectively. Tosashimizu recorded the largest peak value among the 12 stations. Naha and Ishigakijima, located on Okinawa Island, which is southwestern Japan, recorded peals with a smaller amplitude than recorded in the northern area. Both stations observed that the peak began small, and the tsunami wave continued until the largest peak was recorded at 14:00 UTC on 15 January 2022 (23:00 JST). The largest peaks at Naha and Ishigakijima were 0.2 m and 0.1 m, respectively.

The second Lamb wave was measured by atmospheric pressure data and arrived at 00:00 UTC on 17 January 2022, as shown in Fig. 2. The arrival time of the second wave was approximately 44 h after the HTHH eruption and 36 h after the first Lamb wave arrived. The amplitude of the second Lamb was approximately 0.5 hPa, which was smaller than half of the first Lamb wave. Overall, as shown in Fig. 2, tsunami waves gradually increased after the second Lamb wave arrived at approximately 1–2 h. The tsunami height during the early phase was approximately 0.03–0.05 m (see Fig. S3b), and the largest peak occurred in the later phase. The maximum tsunami heights recorded by all stations varied from 0.05 to 0.1 m.

### Wavelet spectral analysis results

**Figure 3:**
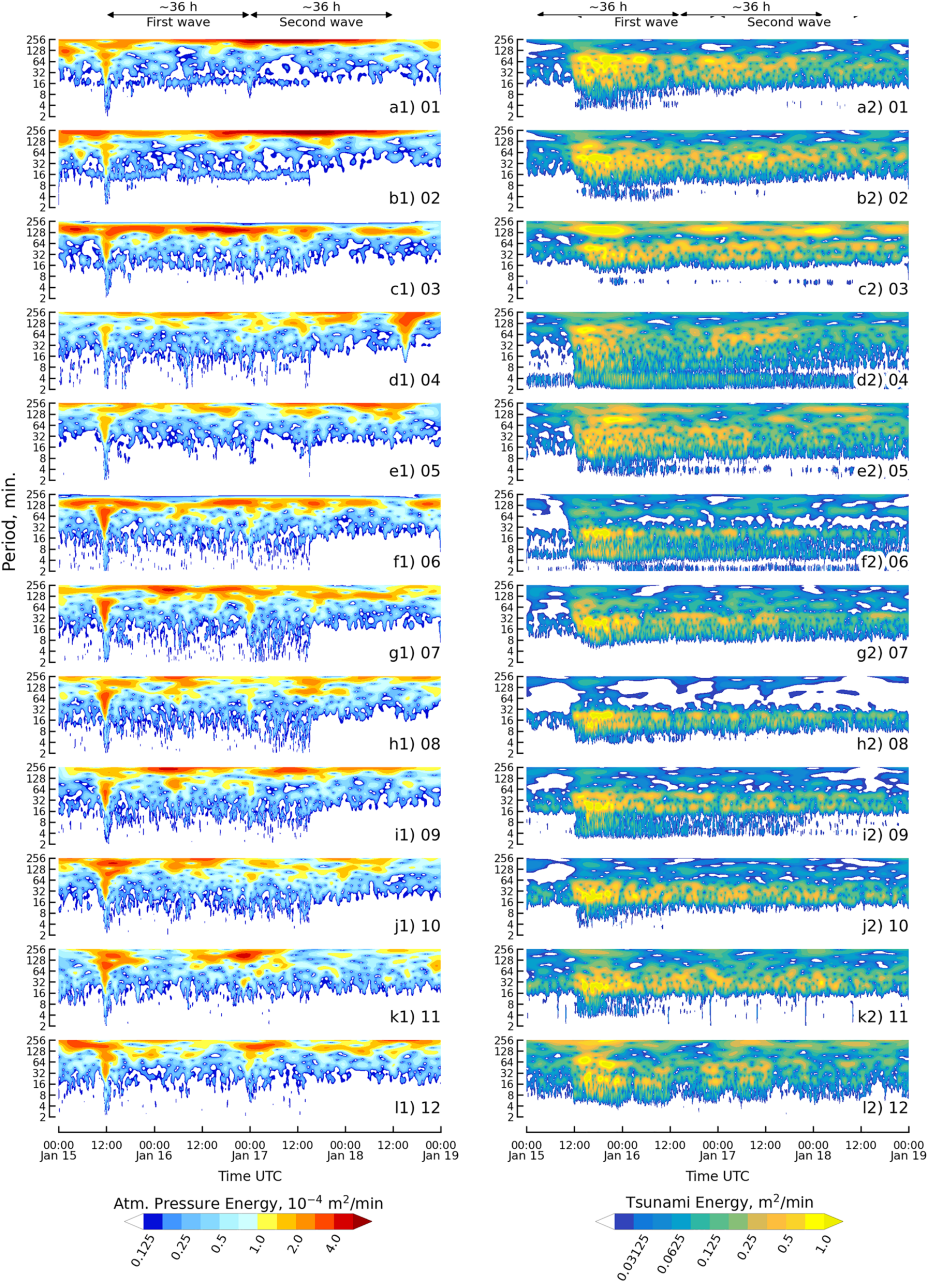
Wavelet spectra of the atmospheric pressure wave and the tsunami wave for the HTHH volcanic eruption as recorded from the 12 observation stations. The first column is the atmospheric pressure wave, and the second column is the tsunami wave.

Wavelet spectra were used to present the duration of the Lamb and tsunami waves generated by the 2022 HTHH eruption. Determining wave duration was not easily accomplished by analyzing the waveform^11^. Figure 3 presents the clear pattern of the duration of two waves (the first and second in the waveform analysis), which are from atmospheric pressure (1st column) and tsunami (2nd column) waves.

The energy signal of the first Lamb wave in the wavelet spectra analysis revealed that the arrival time occurred at 11:00 UTC on 15 January 2022. This result was similar to the findings from the waveform analysis across all stations for the duration of the first wave. The oscillation time of this wave, as recorded by six stations (Hanasaki, Kushiro, Hakodate, Ofunato, Soma, and Ishigakijima), was approximately 3 h, and persisted until 14:00 UTC on 15 January 2022. The main energy was concentrated in the period from 8 to 128 min. The oscillation time was approximately 4 h until 15:00 UTC on 15 January 2022, with the main energy in the period from 4 to 128 min, as recorded by the other six stations, Mera, Omaezaki, Kushimoto, Tosashimizu, Aburatsu, and Naha.

The wavelet analysis of the first tsunami wave reveals that the energy signal had an arrival time of at 11:00 UTC on 15 January 2022, which was similar to the results of the waveform analysis for all stations. The main energy was concentrated in the period from 8 to 128 min for five stations, Hanasaki, Kushiro, Ofunato, Omaezaki, and Naha. The five stations revealed differences in oscillation times of 36 h, 36 h, 18 h, 36 h, and 36 h at Hanasaki, Kushiro, Ofunato, Omaezaki, and Naha, respectively. Two stations (Hakodate and Soma) recorded the main energy in the period range of 8 to 256 min with an oscillating time of approximately 36 h. The main energy of the period ranged from 4 to 64 min at Mera, Kushimoto, and Tosashimizu with oscillating times of 12 h, 36 h, and 24 h, respectively. Aburatsu station had the main energy in the period of 8 to 64 min with an oscillation time longer than 36 h. The main energy in a period of 4 to 256 min was recorded at Ishigakijima station with an oscillating time of 15 h.

For the duration of the second wave, the energy signal of the Lamb wave was identified based on an arrival time at 00:00 UTC on 17 January 2022, which was similar to the results of the waveform analysis. The main energy in the period was in the range from 8 to 128 min, and the energy was lower than the energy in the first wave. The oscillation time of this wave was approximately 2 h. For this second tsunami wave, the energy signal was also lower than the energy signal of the first wave. Eight stations recorded wave energy that continued from the first wave: Hanasaki, Kushiro, Hakodate, Soma, Omaesaki, Kushimoto, Aburatsu, and Naha. The eight stations presented an oscillation time approximately longer than 12 h following the arrival of the second wave. The other four stations provided a lower response signal after the arrival time of the second wave, which was discontinuous with the signal energy of the first wave, Ofunato, Mera, Tosashimizu, and Ishigakijima.

### Fourier spectral analysis results

**Figure 4:**
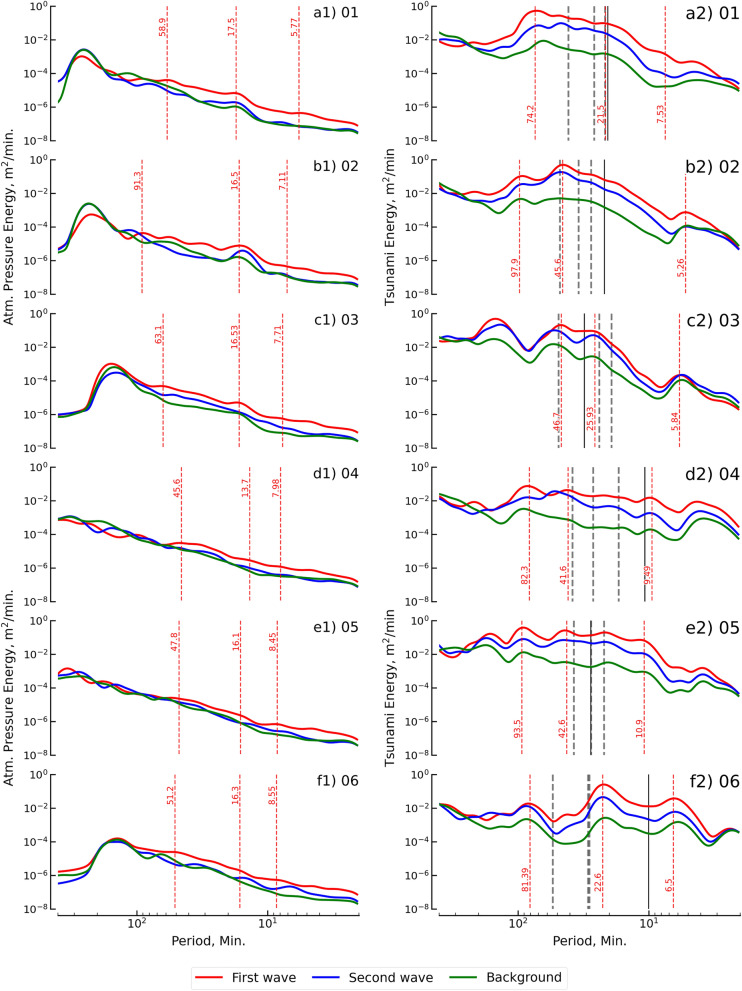

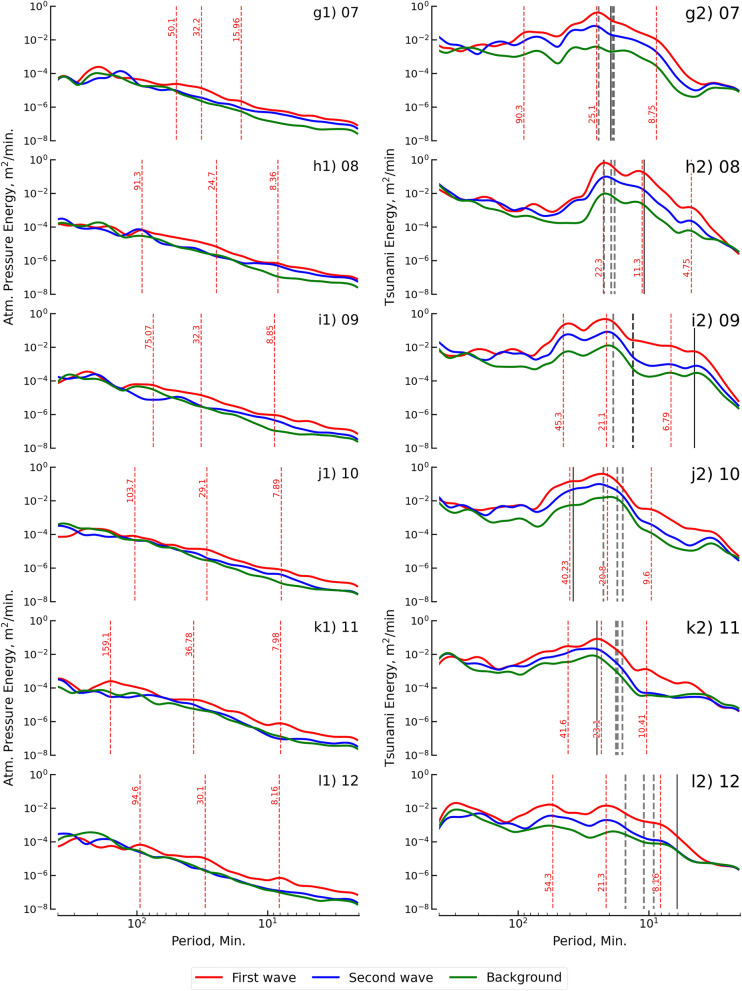
Fourier spectra of the atmospheric pressure wave and the tsunami wave from the 12 stations. Red and blue lines are the spectra of the first and second waves, respectively. The green line is the spectra of the background signals. For the energy of the atmospheric pressure wave, the dominant periods of the first tsunami wave are marked by the vertical line (red dashed line). For the tsunami wave energy, the vertical red dashed line marks the dominant periods. For the natural oscillation within the area around the observation points, the vertical black dashed line represents the period of the eigenmode (regional domain), and the vertical black line is based on the fundamental mode period (local domain). The first column shows the spectra of the atmospheric pressure wave, and the second column shows the spectra of the tsunami wave.

Fourier spectra are used to present the energy spectra related to different periods (or frequencies) in this study. The Fourier spectra of the durations of the first and second waves were plotted and compared with the Fourier spectra of the background waves for atmospheric pressure and tsunami height, as shown in Fig. 4. In Fig. 4, the first column shows the Fourier spectra of atmospheric pressure (Lamb wave), and the second column shows the Fourier spectra of the tsunami height. The gap between wave spectra (first and second waves) and background spectra is the wave energy spectra for the 2022 HTHH eruption. The shapes of the wave spectra are normally followed by the background spectra to reveal the effect of local air pressure and bathymetry on the Lamb and tsunami waves, respectively^11,36,37^.

The Fourier spectra of the Lamb waves were plotted by varying the periods (frequency) from 400 to 2 min (0.0025 to 0.5 Hz) and energies from 1 × 10^–3^ to 1 × 10^–8^ m^2^/min for both wave durations (first and second), as shown in the first column of Fig. 4. The long period (low frequency) energy spectra for both wave durations were greater than 200 min (0.005 Hz) and were close to the energy spectra of the background. The gap in the energy spectra between the Lamb wave spectra and background spectra gradually increased after periods below 200 min for both wave durations. The first wave duration had a gap higher than the second wave duration. Overall, the maximum gaps were located between periods of 5 and 90 min, and the average dominant periods during the gap were approximately 8 to 30 min.

The second column of Fig. 4 shows the Fourier spectra of the tsunami waves that were plotted by the same scale as the Lamb wave (shown in the first column). The energy spectra of both durations for periods greater than 200 min (0.005 Hz) were close to the energy spectra of the background spectra, which was similar to the spectral behavior of the Lamb wave. Additionally, the energy spectra were close to the short period (high frequency) background spectra, which was approximately Below 5 min (0.2 Hz). A period of maximum energy spectra greater than 30 min was observed at five stations in the east, Hansaki, Kushiro, Hakodate, Ofunato, and Soma. The seven stations (Mera, Omaezaki, Kushimoto, Tosashimizu, Aburatsu, Naha, and Ishigakijima) reported a period of maximum energy spectra smaller than 30 min.

**Figure 5:**
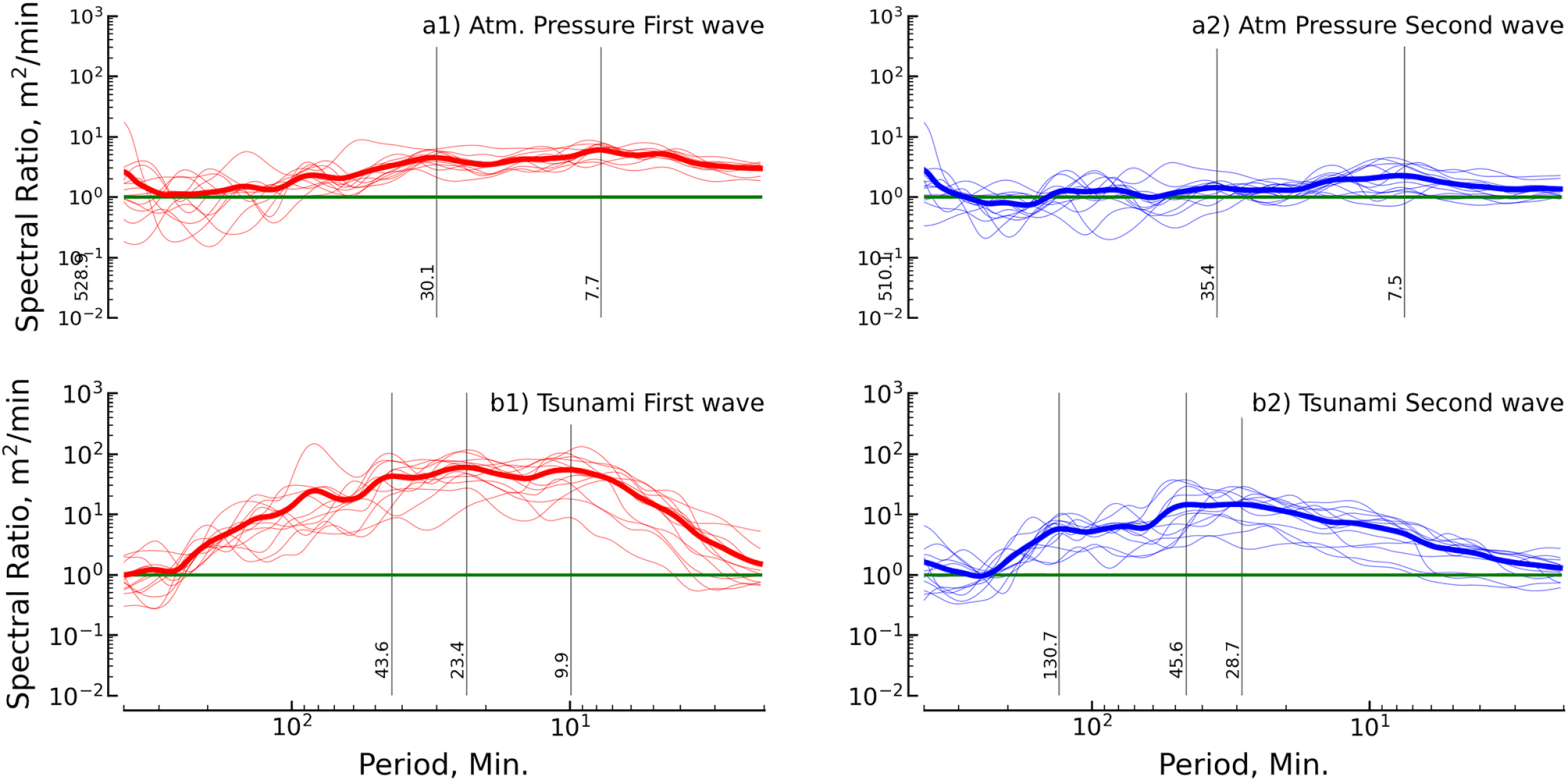
Spectral ratios for the atmospheric pressure wave and tsunami wave. Thin lines are the spectral ratio (ratio between wave spectra and background spectra) for each observation station, and thick lines represent the mean of the 12 stations. The top row of the panel presents the spectral ratio of the atmospheric pressure, and the bottom row panel presents the spectral ratio of the tsunami wave. The first column shows the spectral ratio of the first wave via the red line, and the second column shows the spectral ratio of the second wave in blue.

The source spectrum was assumed by the ratio between the background spectra before and during the tsunami event. The spectral ratios are independent of the local effect on the background condition^36,38^. We estimated the spectral ratio for all stations, as shown in Fig. S5. Figure 5 shows the summary of the spectral ratios of all stations and the mean of all stations with the same sampling rate. Figure 5a1 shows clear energy spectra for the first duration of the atmospheric pressure wave (Lamb wave) in periods ranging from 2 to 150 min. The clear peak in the energy spectra was reported at periods of 7.7 and 30.1 min. The second duration of the Lamb wave showed a clear period in the energy spectra ranging from 2 to 150 min, as shown in Fig. 5a2. The reported peaks of the energy spectra were 7.5 and 35.4 min and were approximately 10 times smaller than peaks for the first duration. The spectral ratio of the first duration of the tsunami wave is presented in Fig. 5b1. The clear energy spectra for the first tsunami duration was a period ranging from 2 to 200 min, with peak energy spectra at periods of 9.9, 23.4, and 34.6 min. Figure 5b2 presents clear energy spectra for the second tsunami duration in the periods of 2 to 200 min. The clear periods of the peak energy spectra were 28.7, 45.6, and 130.7 min. It was concluded by considering the atmospheric pressure data that the energy spectra in periods shorter than 150 min were generated by the Lamb wave. For the tsunami, the energy spectra in a period shorter than 200 min were generated by the tsunami that was triggered by the 2022 HTHH volcanic eruption.

### Modal analysis results

**Figure 6:**
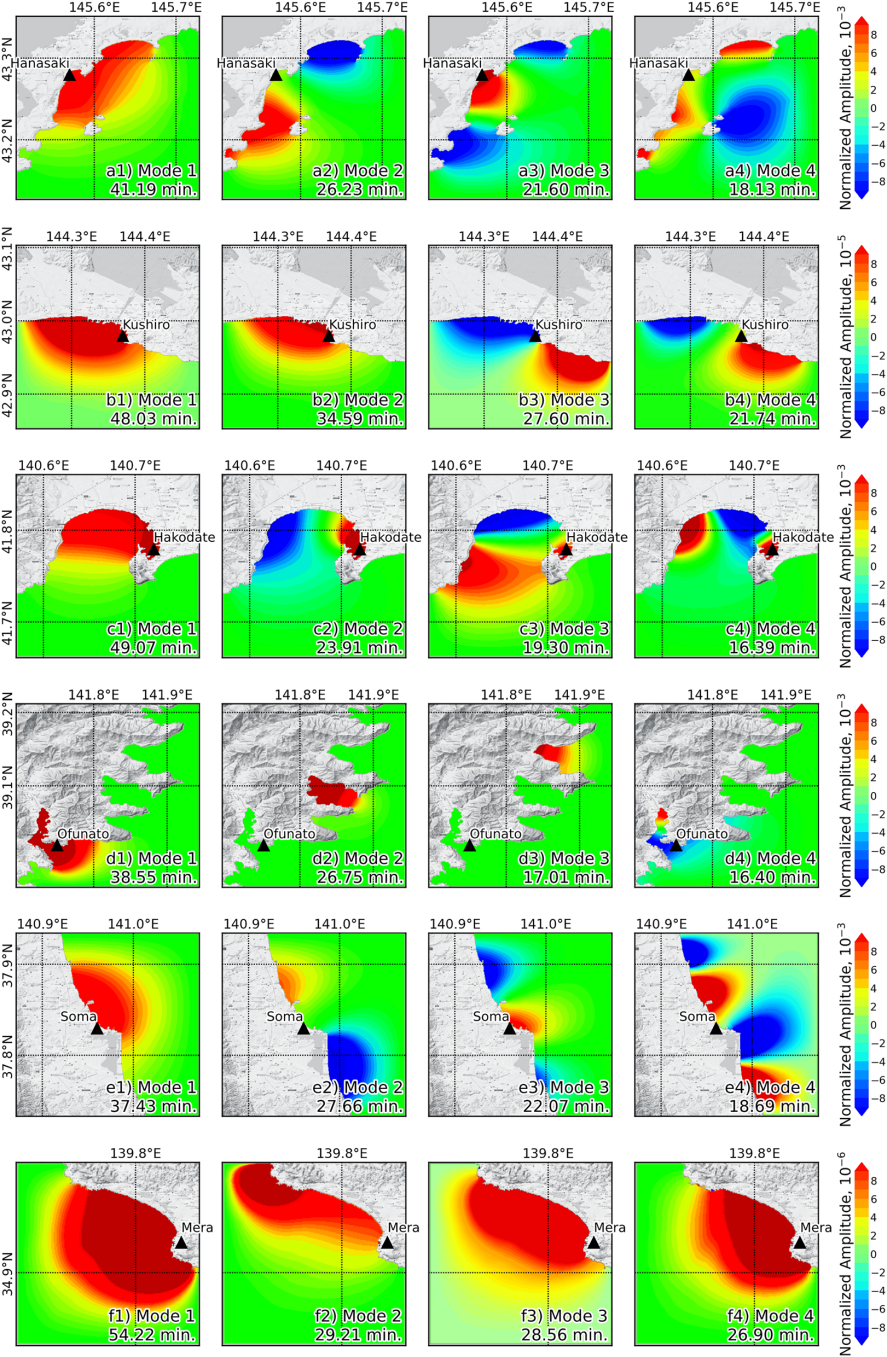

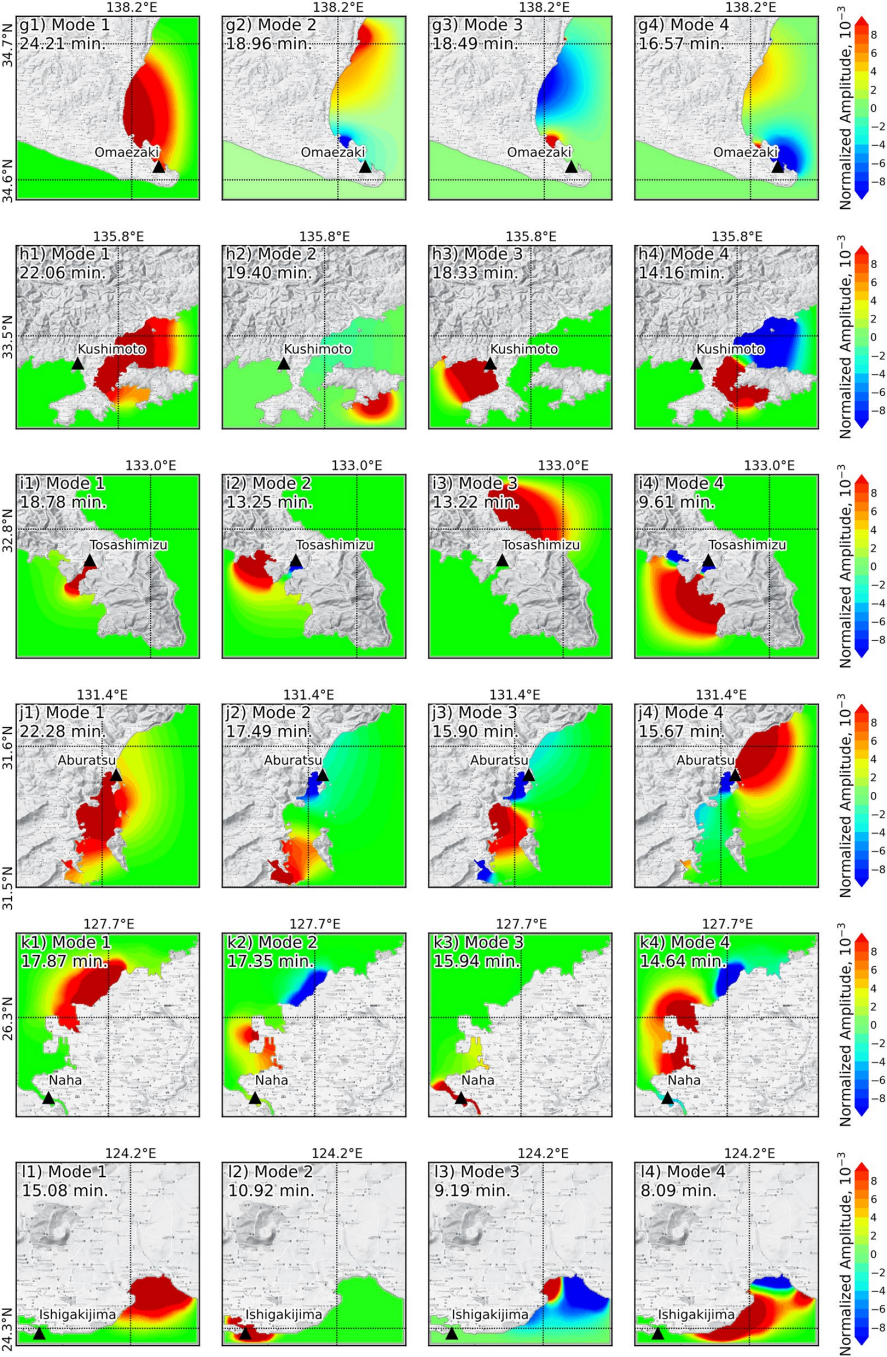
Modal analysis shows representative eigenmodes in the area around each station for the first four modes. The black triangle represents the observation station. The maps were generated using Python version 3.8^66^ with the Matplotlib library^67^, and the basemap was downloaded from the QuickMapServices plugin^68^ through QGIS^60^.

Eigenmodes of regional natural oscillations were calculated by considering the tsunami alone, as shown in Fig. 6. An amplification map of the eigenmode was used to highlight where the zone of increased energy would be concentrated^19,25^. The amplification map is ordered by period. In this study, we plotted the first four eigenmodes that were matched to the tsunami spectral analysis from all selected stations. The periods of the eigenmodes are marked by the vertical lines in Fig. 4 and shown in Table 1. We considered the difference between the periods derived from eigenmodes and from the spectral analysis of temporal data to be less than 10% for increasing amplification. The periods of the first four eigenmodes in the area around the Hanasaki station are 41.19, 23.23, 21.60, and 18.13 min, as shown by the amplification map in Fig. 6a1–a4. Figure 6b1–b4 shows that the periods of the first four eigenmodes in the area around the Kushiro station are 48.03, 34.59, 27.60, and 21.74 min. The mode of the 3rd period from Hanasaki station is close to the dominant periods from the spectral analysis of this station by less than 10%. The mode of the 1st period that is within 10% of the dominant periods from the spectral analysis was reported by six stations, Kushiro, Hakodate, Ofunato, Omaezaki, Kushimoto, and Aburatsu. Additionally, the mode of the 1st period exceeded the dominant period (as determined by spectral analysis) by 10% in Soma, and Tosashimizu stations. However, the 3rd mode from Soma station was within 10% of the dominant period of the spectral analysis. At three stations, Mera, Naha, and Ishigakijima, the eigenmode period did not match the dominant periods of the spectral analysis. The fundamental local mode of oscillation was also calculated for a specific tsunami, as shown in Table 1. All stations are in ports that open to the sea. The periods of the local oscillation modes were almost less than 30 min. There are only two stations where the local oscillation model was longer than 30 min, Hakodate and Naha. At the Hanasaki station, the local oscillation period was close to the dominant period from the spectral analysis, and the 3rd eigenmodes were within 10%. The local oscillation period of Kushimoto station was within 10% of the dominant period from the spectral analysis. At the Aburatsu and Naha stations, the local oscillation period was within 10% of the dominant period but differed the period of the eigenmode by more than 10%. It was concluded that increasing amplification might have been caused by the natural oscillation mode at eight stations: Hanasaki, Kushiro, Hakodate, Ofunato, Omaezaki, Kushimoto, Aburatsu, and Naha.

**Table 1:** Period comparison between the HTHH event (from spectral analysis) and natural oscillation (from regional modal analysis and local fundamental mode analysis) for each area around the observation stations.

Observed station	Spectral analysis, min		Regional modal analysis, min				Local fundamental mode analysis		
			Mode 1	Mode 2	Mode 3	Mode 4	L, m	W, m	Fundamental mode, min
01 Hanasaki	74.2	21.5	41.19	26.23	21.6	18.13	1494	2156	20.61
02 Kushiro	97.9	45.6	48.03	34.59	27.6	21.74	1510	2506	21.91
03 Hakodate	148.4	46.7	49.07	23.91	19.3	16.39	2230	5968	31.23
04 Ofunato	82.3	41.6	38.55	26.75	17.01	16.4	276	250	10.76
05 Soma	93.5	42.6	37.43	27.66	22.07	18.69	1940	4134	27.77
06 Mera	22.6	6.5	54.22	29.21	28.56	26.9	250	320	10.05
07 Omaezaki	90.3	25.1	24.21	18.96	18.49	16.57	1223	2143	19.55
08 Kushimoto	22.3	11.3	22.06	19.4	18.33	14.16	653	660	10.89
09 Tosashimizu	45.3	21.1	18.758	13.25	13.22	9.61	187	506	4.48
10 Aburatsu	45.7	20.8	22.28	17.49	15.9	15.67	2316	1274	37.98
11 Naha	41.6	23.1	17.87	17.35	15.94	14.64	1755	576	24.94
12 Ishigakijima	54.3	21.3	15.08	10.92	9.19	8.09	238	586	6.08

## Conclusion and discussion

The eruption of the HTHH volcano generated atmospheric pressure as the Lamb waves that caused disturbances around the earth^39^. Meteorological stations along the Japanese coastline have measured the Lamb wave multiple times, as shown in Figs. 2 and S2. After the HTHH volcanic eruption, the first Lamb wave arrived after approximately 7 h, as observed from the collected data. The distance from the HTHH volcano to Japan is approximately 8000 km, and, using a sound velocity of approximately 315 m/s^2,40^ reveals that the theoretical arrival time needed for the Lamb wave to reach Japan was approximately 7.1 h. This theoretical arrival time agrees well with the observation data. The shape of the pulse wave from the eruption is an N-wave shape^15,41,42^. N-wave shapes were also present in the observation data for the atmospheric pressure propagation in North America, Australia, New Zealand, and China^1,3,8,11,15,43^. The Lamb waves were also observed at approximately 00:00 UTC on 17 January 2022 approximately 36 h after the arrival time of the first Lamb wave. The second Lamb wave was the reflection of the first Lamb wave considering that the circumference of the Earth is approximately 40,075 km. The amplitude of the second Lamb wave was approximately 4 times smaller than the amplitude of the first Lamb wave. The wavelet spectra analysis also indicated that the first and second arrival times of the Lamb wave were 11:00 UTC on 15 January and 00:00 UTC on 17 January, respectively. There is no evidence that the second Lamb wave was amplified. The source spectra, taken as a ratio between the Lamb wave and the background of the two waves, revealed similar shapes and peak spectra that can be identified as originating from the same source.

The tsunami that impacted the coastline of Japan, as observed from the 12 selected stations, revealed that the arrival time was strongly related to the Lamb wave. The first tsunami wave was generated approximately 2–3 h (approximately 10 h after the HTHH eruption) after the first Lamb wave’s arrival, which was notable for all stations. The second tsunami wave occurred 1 to 2 h after the second Lamb wave arrived, which can be seen in the data. Based on the gravity wave mechanism, the propagation of free waves that emanated from the HTHH volcano toward Japan took approximately 16 h. The observed arrival time from the stations was faster than the times predicted in accordance with the theory of gravity wave, and this was also noted was by Wang et al.^11^ who noted that the wave impacted Linging Bay in China earlier than expected. The wavelet analysis also revealed the tsunami signal approximately 10 h after the HTHH eruption. The wavelet analysis showed the amplification of tsunami height that occurred during the later phase. The source spectra of the tsunami were investigated by taking the spectral ratio of the durations of the two tsunami waves and the background. This revealed that both durations were related to a similar mechanism with similar shapes and peak spectra.

Based on the amplification of tsunami height in the later phase, it is assumed that the dominant period of the tsunami is close to the period of natural oscillation^11,27^. Then, the eigenmodes were used to determine the natural oscillation period. The dominant period of the tsunami was close to the period of natural oscillation at eight stations: Hanasaki, Kushiro, Hakodate, Ofunato, Omaezaki, Kushimoto, Aburatsu, and Naha. The four stations (Ofunato, Kushimoto, Aburatsu, and Naha) that showed that the dominant period was close to the natural period were located in the damaged area, as mentioned by Imamura et al.^12^. This may indicate that the amplification of tsunami resonance was one of the causes of damage in these four areas. The reported damage to marine vessels and aquaculture rafts during the event was a result of resonance^12^. The study’s analysis supports the notion that these four areas align with the reported damage, and the dominant period of the tsunami event was close to the natural oscillation period. Therefore, it may be concluded that the amplification of tsunami resonance played a significant part in the damage incurred within the bay.

The causes of the volcanic tsunami have been explained by several factors. This study’s results provided tsunami characteristics in some observed bays along Japan’s coastline. The tsunami was generated by atmospheric pressure via the Lamb wave that was triggered by the eruption of the HTHH volcano^39^. The results were limited to the affected region within the selected Bays. These results also support the assumption that the tsunami in this event was generated from the Lamb wave. The lead Lamb wave caused the water surface to rise^10^. The rise in water level, which represents the first tsunami wave, occurred concurrently over the deep sea due to the Lamb wave. The first tsunami wave was converted to a gravity wave over the continental slope^10,44^. Then, the maximum height of the first tsunami wave arrived approximately 3–6 h after the Lamb wave impacted Japan’s coastal area. Water subsidence occurred and was converted into total energy after the Lamb wave had passed^7^. The inverse effect of water subsidence generated the gravity wave, which amplified the tsunami energy entering the bay.

This study focuses on using recorded data to study a wave resonance analysis of a tsunami generated by a volcanic explosion’s atmospheric pressure wave (Lamb wave). The study was limited to understanding the tsunami resonance characteristics due to the limited number of observation points in the bay. Using a simplified bay model assumed constant water depth for the local domain, which might not completely represent the complex bathymetry in the bay. Numerical simulations are necessary to improve a comprehensive understanding of the tsunami resonance characteristics in the bay entire. However, simulating this non-seismic tsunami event requires incorporating several unique effects into the numerical tsunami model, including the Earth’s rotation and the atmospheric pressure wave triggered by the volcanic explosion. The interaction between atmospheric pressure and tsunami waves might remain uncertain. Therefore, this study lacks numerical simulation results that could be used for comparison with the analysis results of this study. These limitations highlight the need for the following future study to understand tsunami wave characteristics in a deeper and more accurate representation of this specific event to contribute to tsunami science.

## Methods

### Spectral analysis

We performed two types of spectral analysis on the observed atmospheric pressure and tsunami height data recorded at each station: Wavelet and Fourier analysis. Wavelet analysis is used to determine variations in the dominant peak periods within the time series. The method has been widely applied in wave analysis to understand the temporal variation in wave energy^11,19,45–47^. The adopted wavelet is based on the PyWavelets library in the Python system^48^. The mother function for the applied wavelet was modeled by the Morlet function^11,49,50^. We conducted wavelet analysis to determine the real waveform (atmospheric pressure and tsunami height) of this event, in which the waveform time series was applied for 96 h, to present the spectral energy related to the period and time. Fourier analysis is used to reveal oscillation patterns and frequency behavior^51,52^. In this study, we conducted Fourier analysis to analyze both waveforms (the atmospheric pressure and tsunami height) and to identify their wave energy. The wave energy from the Fourier analysis was proceeded by two intervals, i.e., the wave that impacted during the tsunami and the background conditions, following the visible of atmospheric pressure pulses shown in Fig. 2. The Fourier analysis was conducted using the Numpy library in the Python programming language^53^. The tsunami wave was divided into 2 components, the first wave and the second wave. The first wave component occurred from January 15, 2022, 11:00 UTC to January 17, 2022, 00:00 UTC, and the second wave occurred from January 17, 2022, 00:00 UTC to January 18, 2022, 12:00 UTC. The time series of background component, as derived from the Fourier analysis, occurred from January 14, 2022, 00:00 UTC to January 15, 2022, 11:00 UTC. The background wave spectrum was used to reveal the natural oscillation periods that are a local effect^54^. To remove the local effect, the Lamb wave source spectrum and tsunami source spectrum were calculated by taking the ratio between the wave spectrum during the tsunami and its background spectrum^19,54,55^. In this study, we used this method to present the frequency characteristics of the Lamb wave-generating mechanism and the tsunami-generating mechanism, which is consistent with several previous studies^38,56–58^.

### Modal analysis

The tsunami resonance characteristic is required to determine the dominant periods and their spatial characteristics when investigating the eigenmodes of natural oscillations^59^. The eigenmodes of natural oscillations are based on bathymetry data. The bathymetry data used were resampled with a resolution of 1.6 arc-second by using the cubic spline method in QGIS^60^. The bathymetry data were obtained from the Association for Promotion of Infrastructure Geospatial Information Distribution of Japan^61^. We investigated the tsunami resonance characteristics based on 2 domains, regional and local. The regional domains were used to provide information on large-scale modes in the area (10–30 km) that covers the bay where the selected observed stations were located, as shown in detail in Fig. S6. The local domains were used to provide information on smaller-scale modes, such as in the area (1–3 km) that covers the port in the bay for the regional domains, as shown in detail in Fig. S7. We calculated the eigenmodes of the natural oscillation in each selected bay (regional domain) using our developed numerical model that a governing equation is originally from Loomis^62^. The numerical model required only the bathymetry data. This model was solved using the linear solution to the shallow water equation (SWE) in a staggered grid system on the finite difference method. The governing equation of the model is as follows:

1 $$\nabla \cdot \left(h\Delta \varnothing \right)=\frac{1}{g}\cdot \frac{{\partial }^{2}\varnothing }{{\partial t}^{2}}$$

where ${\varphi }_{x,y,t}$ is the water level, ${h}_{x,y}$ is the water depth, and *g* is the gravitational acceleration (9.81 m/s^2^). The coastal boundary was assumed to be fully reflective and was without flux, and the open sea boundary was assumed to have no reflection conditions. The coordinate system of the original model is based on the Cartesian system that is unsuitable for depicting the actual surface of the earth. Therefore, we modified the governing equation of the original model to a spherical coordinate system based on Wu and Satake^63^. The differential equation given in Eq. (1), in the form of a matrix-eigenvalue problem, was modified to spherical coordinates in this study, as shown by Eq. (2):

2 $$\frac{1}{{R}^{2\mathrm{cos}\theta }}\frac{\partial }{\partial \theta }\left(h\mathrm{cos}\theta \frac{\partial \varnothing }{\partial \theta }\right)+\frac{1}{{R}^{2}\mathrm{cos}\theta }\left(h\frac{\partial \varnothing }{\partial \varphi }\right)=\lambda \varnothing $$

where $\theta$ is the latitude, $\varphi$ is the longitude, *R* is the Earth’s radius, and *λ* = *ω*^2^*/g* is the eigenvalue. The detail of the numerical method for this model was presented in supplementary S1.

We applied the method proposed by Rabinovich^64^ and Ivanov et al.^65^ to estimate the natural oscillation modes of the port considered for the local domain, which is where the selected stations were located. All stations were located in ports that face the open sea. In the method used for studying tsunami resonance in the port, the period of oscillation mode is determined for a rectangular basin with a constant water depth, which can be expressed as:

3 $${T}_{c}=\frac{4LW}{\sqrt{gh{(1+2k)}^{2}{W}^{2}+4{m}^{2}{L}^{2}}}; \quad k,m=\mathrm{0,1},2,\dots $$

where *L* is the length of the port measured in the direction perpendicular to the port entrance and *W* is the width of the port parallel to the entrance. The details of both dimensions are shown in the left panel of Figs. S7 and 1. *h* is the average water depth in the port selected by the maximum probability based on the histogram, and the details used to estimate this variable are shown in the right panel of Fig. S7. In this study, we considered only the fundamental mode with *k* = 0 and *m* = 0. We compared the calculated eigenmode of regional and local domains with the dominant period of the spectral analysis. It is possible that damage was due to the increased amplification caused by periods that deviate by less than 10% from the period of eigenmodes, as inferred from spectral analysis of the time series^19,27^.

### Supplementary Information

Supplementary Figures.

## Data Availability

The atmospheric pressure data used in this study are from Weathernews Inc. (https://global.weathernews.com/news/16551/). The sea water level data is available from Intergovernmental Oceanographic Commission (http://www.ioc-sealevelmonitoring.org) and Geospatial Information Authority of Japan (https://www.gsi.go.jp/kanshi/tide_furnish.html). The bathymetry data was provided from Association for Promotion of Infrastructure Geospatial Information Distribution of Japan (https://front.geospatial.jp/). The code for Wavelet analysis can be downloaded from PyWavelets (https://pywavelets.readthedocs.io/). The code for Fourier analysis is availed from Numpy (https://numpy.org/doc/stable/reference/routines.fft.html).

## Code availability

The code that supports the findings in this study are available from the corresponding author upon resonable request.

## References

[1] Amores A (2022). Numerical simulation of atmospheric lamb waves generated by the 2022 Hunga-Tonga volcanic eruption. Geophys. Res. Lett..

[2] Nishida K, Kobayashi N, Fukao Y (2014). Background lamb waves in the earth’s atmosphere. Geophys. J. Int..

[3] Gusman AR (2022). The 2022 Hunga Tonga-Hunga Ha’apai volcano air-wave generated tsunami. Pure Appl. Geophys..

[4] Monserrat S, Vilibić I, Rabinovich AB (2006). Meteotsunamis: Atmospherically induced destructive ocean waves in the tsunami frequency band. Nat. Hazard. Earth Syst. Sci..

[5] Bechle AJ, Wu CH (2014). The lake Michigan meteotsunamis of 1954 revisited. Nat. Hazards.

[6] Ramírez-Herrera M, Coca O, Vargas-Espinosa V (2022). Tsunami effects on the coast of mexico by the Hunga Tonga-Hunga Ha’apai volcano eruption, Tonga. Pure Appl. Geophys..

[7] Kubota T, Saito T, Nishida K (2022). Global fast-traveling tsunamis by atmospheric pressure waves on the 2022 Tonga eruption. Science.

[8] Ortiz-Huerta LG, Ortiz M (2022). On the Hunga-Tonga complex tsunami as observed along the pacific coast of Mexico on January 15, 2022. Pure Appl. Geophys..

[9] Manneela S, Kumar S (2022). Overview of the Hunga Tonga-Hunga Ha’apai volcanic eruption and tsunami. J. Geol. Soc. India..

[10] Tanioka Y, Yamanaka Y, Nakagaki T (2022). Characteristics of the deep sea tsunami excited offshore Japan due to the air wave from the 2022 Tonga eruption. Earth. Planet Space..

[11] Wang Y, Wang P, Kong H, Wong CS (2022). Tsunamis in Lingding Bay, China, caused by the 2022 Tonga volcanic eruption. Geophys. J. Int..

[12] Imamura F (2022). Preliminary observations and impact in Japan of the tsunami caused by the Tonga volcanic eruption on January 15. Pure Appl. Geophys..

[13] Wang Y (2023). Data assimilation using high-frequency radar for tsunami early warning: A case study of the 2022 Tonga volcanic tsunami. J. Geophys. Res. Solid Earth.

[14] Wang Y, Imai K, Mulia IE, Kusumoto S, Takahashi N (2022). Tsunami early warning of the Hunga volcanic eruption using an ocean floor observation network off the Japanese islands. Seismol. Res. Lett..

[15] Lynett P (2022). Diverse tsunamigenesis triggered by the Hunga Tonga-Hunga Ha’apai eruption. Nature.

[16] La Selle, S. M. *et al.* Observations of tsunami and runup heights in santa cruz harbor and surrounding beaches from the 2022 Hunga Tonga-Hunga Ha’apai tsunami, Doi: 10.5066/P9ZVAB8D (2022).

[17] INDECI. Inician acciones de respuesta luego de oleajes en el litoral. retrieved. *Retrieved* https://www.gob.pe/institucion/indeci/noticias/576687-inician-acciones-de-respuesta-luego-de-oleajes-en-el-litoral (2022).

[18] UNOCHA. Peru: Oil spill, flash update no. 02. retrieved. *Retrieved* https://reliefweb.int/report/peru/peru-oil-spill-flash-update-no-02-27-january-2022 (2022).

[19] Wang Y, Zamora N, Quiroz M, Satake K, Cienfuegos R (2021). Tsunami resonance characterization in Japan due to trans-pacific sources: Response on the bay and continental shelf. J. Geophys. Res. Ocean..

[20] Allgeyer S, Hébert H, Madariaga R (2013). Modeling the tsunami free oscillations in the marquesas (French Polynesia). Geophys. J. Int..

[21] Heidarzadeh M, Satake K (2014). Excitation of basin-side modes of the pacific ocean following the March 2011 Tohoku tsunami. Pure Appl. Geophys..

[22] Wilson BW (1972). Modeling the tsunami free oscillations in the marquesas (French polynesia). Adv. Hydrosci..

[23] Okal EA (2006). Oman field survey after the December 2004 Indian ocean tsunami. Earthq. Spectra.

[24] Munger S, Cheung KF (2008). Resonance in hawaii waters from the 2006 kuril islands tsunami. Geophys. Res. Lett..

[25] Yamazaki Y, Cheung K (2011). Shelf resonance and impact of near filed tsunami generated by the 2010 chile earthquake. Geophys. Res. Lett..

[26] Zhao X, Liu H (2017). Resonance of long wave runup on south China sea’s piecewise topographies. J. Earthq. Tsunami..

[27] Cortés P, Catalán P, Aránguiz R, Bellotti G (2017). Tsunami and shelf resonance on the northern chile coast. J. Geophys. Res..

[28] Abe K (2005). Tsunami resonance curve from dominant periods observed in bays of northeastern japan. Adv. Nat. Technol. Hazards Res..

[29] Inc., W. Atmospheric pressure data from soratena weather sensors offered to researhers free of charge. retrieved. *Retrieved* https://global.weathernews.com/news/16551/ (2022).

[30] Haurwitz B, Cowley AD (1973). The diurnal and semidiurnal barometric oscillations global distribution and annual variation. Pure Appl. Geophys..

[31] VLIZ. Flanders marine institute, intergovernmental oceanographic commission (IOC) (2022): Sea level station monitoring facility. *Accessed* http://www.ioc-sealevelmonitoring.org (2022).

[32] Geospatial Information Authority of Japan, G. Geospatial information authority of japan. *Accessed* https://www.gsi.go.jp/kanshi/tide_furnish.html (2022).

[33] Pakoksung K, Suppasri A, Imamura F (2022). The near-field tsunami generated by the 15 January 2022 eruption of the Hunga Tonga-Hunga Ha’apai volcano and its impact on Tongatapu, Tonga. Sci Rep..

[34] Heidarzadeh M, Satake K (2013). Waveform and spectral analyses of the 2011 Japan tsunami records on tide gauge and dart stations across the Pacific ocean. Pure Appl. Geophys..

[35] Ren Z, Hou J, Wang P, Wang Y (2021). Tsunami resonance and standing waves in Hangzhou bay. Phys. Fluids.

[36] Rabinovich AB, Titov VV, Moore CW, Eblé MC (2017). The 2004 Sumatra tsunami in the southeastern Pacific ocean: New global insight from observations and modeling. J. Geophys. Res. Ocean..

[37] Heidarzadeh M, Satake K (2014). The El Salvador and Philippines tsunamis of August 2012: Insights from sea level data analysis and numerical modeling. Pure Appl. Geophys..

[38] Toguchi Y, Fujii S, Hinata H (2018). Tsunami waves and tsunami-induced natural oscillations determined by HF radar in Ise bay, Japan. J. Geophys. Res. Ocean..

[39] Nishikawa Y (2022). Observation and simulation of atmospheric gravity waves exciting subsequent tsunami along the coastline of Japan after Tonga explosion event. Sci. Rep..

[40] Abbrescia M (2022). Observation of Rayleigh-Lamb waves generated by the 2022 Hunga-Tonga volcanic eruption with the pola detectors at ny-Ålesund. Sci. Rep..

[41] Fitzgerald TJ (1997). Observations of total electron content perturbations on GPS signals caused by a ground level explosion. J. Atmos. Sol. Terr. Phys..

[42] Matoza RS (2022). Atmospheric waves and global seismoacoustic observations of the January 2022 Hunga eruption, Tonga. Science.

[43] Hu G, Li L, Ren Z, Zhang K (2022). The characteristics of the 2022 Tonga volcanic tsunami in the pacific ocean. Nat. Hazards Earth Syst. Sci..

[44] Tonegawa T, Fukao Y (2022). Wave propagation of meteotsunamis and generation of free tsunamis in the sloping area of the japan trench for the 2022 Hunga–Tonga volcanic eruption. Earth Planets Space..

[45] Nagarajan B (2006). The great tsunami of 26 December 2004: A description based on tide-gauge data from the Indian subcontinent and surrounding areas. Earth Planets Space..

[46] Heidarzadeh M, Satake K (2013). The 21 May 2003 tsunami in the western mediterranean sea: Statistical and wavelet analyses. Pure Appl. Geophys..

[47] Vilibić I, Šepić J (2017). Global mapping of nonseismic sea level oscillations at tsunami timescales. Sci. Rep..

[48] Lee GR, Gommers R, Wasilewski F, Wohlfahrt K, O’Leary A (2019). Pywavelets: A python package for wavelet analysis. J. Open Source Softw..

[49] Goupillaud P, Grossmann A, Morlet J (1984). Cycle-octave and related transforms in seismic signal analysis. Geoexploration.

[50] Torrence C, Compo GP (1998). A practical guide to wavelet analysis. Bull. Am. Meteorol. Soc..

[51] Ren Z, Liu H, Jimenez C, Wang Y (2022). Tsunami resonance and standing waves in the south China sea. Ocean. Eng..

[52] Catalán PA (2015). The 1 April 2014 Pisagua tsunami: Observations and modeling. Geophys. Res. Lett..

[53] Harris CR (2020). Array programming with numpy. Nature.

[54] Rabinovich B (1997). Spectral analysis of tsunami waves: Separation of source and topography effects. J. Geophys. Res..

[55] Heidarzadeh M, Harada T, Satake K, Ishibe T, Gusman AR (2016). Comparative study of two tsunamigenic earthquakes in the solomon islands: 2015 mw 7.0 normal-fault and 2013 santa cruz mw 8.0 megathrust earthquakes. Geophys. Res. Lett..

[56] Vich M, Monserrat S (2003). Source spectrum for the Algerian tsunami of 21 May 2003 estimated from coastal tide gauge data. Geophys. Res. Lett..

[57] Zaytsev O, Rabinovich AB, Thomson RE (2016). A comparative analysis of coastal and open-ocean records of the great chilean tsunamis of 2010, 2014 and 2015 off the coast of mexico. Pure Appl. Geophys..

[58] Rabinovich AB, Fritz HM, Tanioka Y, Geist EL (2017). Global tsunami science: Past and future. Pure Appl. Geophys..

[59] Bellotti G, Briganti R, Beltrami GM (2012). The combined role of bay and shelf modes in tsunami amplification along the coast. J. Geophys. Res. Ocean..

[60] QGIS. Qgis geographic information system. *QGIS Association* http://www.qgis.org (2022).

[61] Geospatial. Association for promotion of infrastructure geospatial information distribution of japan. *Geospatial* https://front.geospatial.jp/ (2023).

[62] G., L. H. Normal modes of oscillation of honokohau harbor. (noaa-jtre-142, honolulu, hi: Hawaii institute of geophysics, university of hawaii. *Loomis* https://books.google.co.jp/books?id=3_F2OwAACAAJ (1975).

[63] Wu Y, Satake K (2018). Synthesis and source characteristics of tsunamis in the sea of Japan based on normal-mode method. J. Geophys. Res. Solid Earth..

[64] Rabinovich AB, Kim YC (2010). Seiches and harbor oscillations. Handb. Coast. Ocean. Eng..

[65] Ivanov VA, Manilyuk YV, Sannikov VF (2018). Seiches in a basin with an open entrance. J. Appl. Mech. Tech. Phys..

[66] van Rossum, G. & Drake, F. L. Python 3 reference manual. *Loomis* http://www.python.org (Createspace, 2009).

[67] Hunter JD (2018). A 2D graphics environment. Comput. Sci. Eng..

[68] NextGIS. Quickmapservices: Easy basemaps in qgis. *Loomis* https://nextgis.com/blog/quickmapservices/ (2019).

