# Supplementary material for: Resonance characteristics of tsunami in bay of Japan by the Hunga Tonga-Hunga Ha’apai volcano eruption on 15th January 2022
Source: Sci Rep. 2023 Oct 26;13:18385. doi: 10.1038/s41598-023-45601-6 (PMC10603173; doi:10.1038/s41598-023-45601-6)
Supplement: Supplementary file 1 — Supplementary Figures. [file 41598_2023_45601_MOESM1_ESM.pdf]

Resonance Characteristics of Tsunami in Bay of Japan by the Hunga Tonga-Hunga Ha'apai Volcano Eruption on 15th January 2022

Supplementary document 1

Kwanchai Pakoksung[1,*], Anawat Suppasri[1], and Fumihiko Imamura[1]

[1] *International Research Institute of Disaster Science, Tohoku University, Sendai, 980-0845, Japan*
[*] *pakoksung@irides.tohoku.ac.jp*

Figure list

Figure S1 Atmospheric pressure waveform from the **Hanasaki** observation point. a) Raw waveform data and low-frequency trend (from the high-pass filter), b) Filtered waveform data from the high-pass filter.
Figure S1 (Cont.) Atmospheric pressure waveform from the **Kushiro** observation point. a) Raw waveform data and low-frequency trend (from the high-pass filter), b) Filtered waveform data from the high-pass filter.
Figure S1 (Cont.) Atmospheric pressure waveform from the **Hakodate** observation point. a) Raw waveform data and low-frequency trend (from the high-pass filter), b) Filtered waveform data from the high-pass filter..
Figure S1 (Cont.) Atmospheric pressure waveform from the **Ofunato** observation point. a) Raw waveform data and low-frequency trend (from the high-pass filter), b) Filtered waveform data from the high-pass filter.
Figure S1 (Cont.) Atmospheric pressure waveform from the **Soma** observation point. a) Raw waveform data and low-frequency trend (from the high-pass filter), b) Filtered waveform data from the high-pass filter.
Figure S1 (Cont.) Atmospheric pressure waveform from the **Mera** observation point. a) Raw waveform data and low-frequency trend (from the high-pass filter), b) Filtered waveform data from the high-pass filter.
Figure S1 (Cont.) Atmospheric pressure waveform from the **Omaezaki** observation point. a) Raw waveform data and low-frequency trend (from the high-pass filter), b) Filtered waveform data from the high-pass filter.
Figure S1 (Cont.) Atmospheric pressure waveform from the **Kushimoto** observation point. a) Raw waveform data and low-frequency trend (from the high-pass filter), b) Filtered waveform data from the high-pass filter.
Figure S1 (Cont.) Atmospheric pressure waveform from the **Tosashimizu** observation point. a) Raw waveform data and low-frequency trend (from the high-pass filter), b) Filtered waveform data from the high-pass filter.
Figure S1 (Cont.) Atmospheric pressure waveform from the **Aburatsu** observation point. a) Raw waveform data and low-frequency trend (from the high-pass filter), b) Filtered waveform data from the high-pass filter.
Figure S1 (Cont.) Atmospheric pressure waveform from the **Naha** observation point. a) Raw waveform data and low-frequency trend (from the high-pass filter), b) Filtered waveform data from the high-pass filter.
Figure S1 (Cont.) Atmospheric pressure waveform from the **Ishigakijima** observation point. a) Raw waveform data and low-frequency trend (from the high-pass filter), b) Filtered waveform data from the high-pass filter.
Figure S2 Time series of atmospheric pressure records from the 12 stations. The largest

explosion from HTHH occurred on 04:14:45 UTC, 15 January 2022, and is marked with a red dashed line. The first (12:00 UTC, 15 January 2022 to 00:00 UTC, 17 January 2022) and second (00:00 UTC, 17 January 2022 to 12:00 UTC, 18 January 2022) wave components were approximately 36 hours apart.

Figure S3 The tsunami waveform from the **Hanasaki** observation point. a) Raw waveform data and low-frequency trend (from the high-pass filter), b) Filtered waveform data from the high-pass filter.

Figure S3 (Cont.) The tsunami waveform from the **Kushiro** observation point. a) Raw waveform data and low-frequency trend (from the high-pass filter), b) Filtered waveform data from the high-pass filter.

Figure S3 (Cont.) The tsunami waveform from the **Hakodate** observation point. a) Raw waveform data and low-frequency trend (from the high-pass filter), b) Filtered waveform data from the high-pass filter..

Figure S3 (Cont.) The tsunami waveform from the **Ofunato** observation point. a) Raw waveform data and low-frequency trend (from the high-pass filter), b) Filtered waveform data from the high-pass filter.

Figure S3 (Cont.) The tsunami waveform from the **Soma** observation point. a) Raw waveform data and low-frequency trend (from the high-pass filter), b) Filtered waveform data from the high-pass filter.

Figure S3 (Cont.) The tsunami waveform from the **Mera** observation point. a) Raw waveform data and low-frequency trend (from the high-pass filter), b) Filtered waveform data from the high-pass filter.

Figure S3 (Cont.) The tsunami waveform from the **Omaezaki** observation point. a) Raw waveform data and low-frequency trend (from the high-pass filter), b) Filtered waveform data from the high-pass filter.

Figure S3 (Cont.) The tsunami waveform from the **Kushimoto** observation point. a) Raw waveform data and low-frequency trend (from the high-pass filter), b) Filtered waveform data from the high-pass filter.

Figure S3 (Cont.) The tsunami waveform from the **Tosashimizu** observation point. a) Raw waveform data and low-frequency trend (from the high-pass filter), b) Filtered waveform data from the high-pass filter.

Figure S3 (Cont.) The tsunami waveform from the **Aburatsu** observation point. a) Raw waveform data and low-frequency trend (from the high-pass filter), b) Filtered waveform data from the high-pass filter.

Figure S3 (Cont.) The tsunami waveform from the **Naha** observation point. a) Raw waveform data and low-frequency trend (from the high-pass filter), b) Filtered waveform data from the high-pass filter.

Figure S3 (Cont.) The tsunami waveform from the **Ishigakijima** observation point. a) Raw waveform data and low-frequency trend (from the high-pass filter), b) Filtered waveform data from the high-pass filter.

Figure S4 Time series of sea surface elevation records from the 12 stations. The largest explosion of HTHH occurred on 04:14:45 UTC, 15 January 2022, and is indicated by a red dashed line. The first (12:00 UTC, 15 January 2022 to 00:00 UTC, 17 January 2022) and second (00:00 UTC, 17 January 2022 to 12:00 UTC, 18 January 2022) wave components were separated by approximately 36 hours.

Figure S5 Spectra ratios of the atmospheric pressure wave and tsunami wave for the **Hanasaki** station. Red lines are the ratio between the wave spectra and background spectra. The top row presents the spectral ratio of the atmospheric pressure, and the bottom row presents the spectral ratio of the tsunami wave. The first column presents the spectral ratio of the first wave, and the second column presents the spectral ratio of the second wave.

Figure S5 (Cont.) Spectra ratios of the atmospheric pressure wave and tsunami wave for the **Kushiro** station. Red lines are the ratio between the wave spectra and background spectra. The top row presents the spectral ratio of the atmospheric pressure, and the bottom row presents the spectral ratio of the tsunami wave. The first column presents the spectral ratio of the first wave, and the second column presents the spectral ratio of the second wave.

Figure S5 (Cont.) Spectra ratios of the atmospheric pressure wave and tsunami wave for the **Hakodate** station. Red lines are the ratio between the wave spectra and background

spectra. The top row presents the spectral ratio of the atmospheric pressure, and the bottom row presents the spectral ratio of the tsunami wave. The first column presents the spectral ratio of the first wave, and the second column presents the spectral ratio of the second wave.

Figure S5 (Cont.) Spectra ratios of the atmospheric pressure wave and tsunami wave for the **Ofunato** station. Red lines are the ratio between the wave spectra and background spectra. The top row presents the spectral ratio of the atmospheric pressure, and the bottom row presents the spectral ratio of the tsunami wave. The first column presents the spectral ratio of the first wave, and the second column presents the spectral ratio of the second wave.

Figure S5 (Cont.) Spectra ratios of the atmospheric pressure wave and tsunami wave for the **Soma** station. Red lines are the ratio between the wave spectra and background spectra. The top row presents the spectral ratio of the atmospheric pressure, and the bottom row presents the spectral ratio of the tsunami wave. The first column presents the spectral ratio of the first wave, and the second column presents the spectral ratio of the second wave.

Figure S5 (Cont.) Spectra ratios of the atmospheric pressure wave and tsunami wave for the **Mera** station. Red lines are the ratio between the wave spectra and background spectra. The top row presents the spectral ratio of the atmospheric pressure, and the bottom row presents the spectral ratio of the tsunami wave. The first column presents the spectral ratio of the first wave, and the second column presents the spectral ratio of the second wave.

Figure S5 (Cont.) Spectra ratios of the atmospheric pressure wave and tsunami wave for the **Omaezaki** station. Red lines are the ratio between the wave spectra and background spectra. The top row presents the spectral ratio of the atmospheric pressure, and the bottom row presents the spectral ratio of the tsunami wave. The first column presents the spectral ratio of the first wave, and the second column presents the spectral ratio of the second wave.

Figure S5 (Cont.) Spectra ratios of the atmospheric pressure wave and tsunami wave for the **Kushimoto** station. Red lines are the ratio between the wave spectra and background spectra. The top row presents the spectral ratio of the atmospheric pressure, and the bottom row presents the spectral ratio of the tsunami wave. The first column presents the spectral ratio of the first wave, and the second column presents the spectral ratio of the second wave.

Figure S5 (Cont.) Spectra ratios of the atmospheric pressure wave and tsunami wave for the **Tosashimizu** station. Red lines are the ratio between the wave spectra and background spectra. The top row presents the spectral ratio of the atmospheric pressure, and the bottom row presents the spectral ratio of the tsunami wave. The first column presents the spectral ratio of the first wave, and the second column presents the spectral ratio of the second wave.

Figure S5 (Cont.) Spectra ratios of the atmospheric pressure wave and tsunami wave for the **Aburatsu** station. Red lines are the ratio between the wave spectra and background spectra. The top row presents the spectral ratio of the atmospheric pressure, and the bottom row presents the spectral ratio of the tsunami wave. The first column presents the spectral ratio of the first wave, and the second column presents the spectral ratio of the second wave.

Figure S5 (Cont.) Spectra ratios of the atmospheric pressure wave and tsunami wave for the **Naha** station. Red lines are the ratio between the wave spectra and background spectra. The top row presents the spectral ratio of the atmospheric pressure, and the bottom row presents the spectral ratio of the tsunami wave. The first column presents the spectral ratio of the first wave, and the second column presents the spectral ratio of the second wave.

Figure S5 (Cont.) Spectra ratios of the atmospheric pressure wave and tsunami wave for the **Ishigakijima** station. Red lines are the ratio between the wave spectra and background spectra. The top row presents the spectral ratio of the atmospheric pressure, and the bottom row presents the spectral ratio of the tsunami wave. The first column presents the spectral ratio of the first wave, and the second column presents the spectral ratio of the second wave.

Figure S6 Bathymetry data for the regional domain that covers the bay where the

selected observation stations are located. Contours are in 10 m intervals. The maps were generated using Python version 3.8 (http://www.python.org) with the Matplotlib library (https://matplotlib.org/), and the basemap was downloaded from the QuickMapServices plugin (https://nextgis.com/blog/quickmapservices/) through QGIS (http://www.qgis.org).

Figure S7 Left panel) Bathymetry data for the regional domain that covers the bay where the selected observation stations are located. Contours are marked in 2.5 m intervals. The approximated geometry of the port is represented by the yellow rectangle, which was used to estimate the local oscillation modes. The maps were generated using Python version 3.8 (http://www.python.org) with the Matplotlib library (https://matplotlib.org/), and the basemap was downloaded from the QuickMapServices plugin (https://nextgis.com/blog/quickmapservices/) through QGIS (http://www.qgis.org). Right panel) A histogram of water depth in the port was used to identify the representative port depth to estimate the local oscillations.

Figure S7 (Cont.) Left panel) Bathymetry data for the regional domain that covers the bay where the selected observation stations are located. Contours are marked in 2.5 m intervals. The approximated geometry of the port is represented by the yellow rectangle, which was used to estimate the local oscillation modes. The maps were generated using Python version 3.8 (http://www.python.org) with the Matplotlib library (https://matplotlib.org/), and the basemap was downloaded from the QuickMapServices plugin (https://nextgis.com/blog/quickmapservices/) through QGIS (http://www.qgis.org). Right panel) A histogram of water depth in the port was used to identify the representative port depth to estimate the local oscillations.

Figure S7 (Cont.) Left panel) Bathymetry data for the regional domain that covers the bay where the selected observation stations are located. Contours are marked in 2.5 m intervals. The approximated geometry of the port is represented by the yellow rectangle, which was used to estimate the local oscillation modes. The maps were generated using Python version 3.8 (http://www.python.org) with the Matplotlib library (https://matplotlib.org/), and the basemap was downloaded from the QuickMapServices plugin (https://nextgis.com/blog/quickmapservices/) through QGIS (http://www.qgis.org). Right panel) A histogram of water depth in the port was used to identify the representative port depth to estimate the local oscillations.

S1. Discretization of Modal analysis for the regional area

The linear solution on the shallow water equation (SWE) from Equation 1 in the main text is as follows:

$$\bigtriangledown \cdot (h \bigtriangledown \phi) = \frac{1}{g}\frac{\partial^2 \phi}{\partial t^2} \tag{S1}$$

where $\phi_{x,y,t}$ is the water level, $h_{x,y}$ is the water depth, and g is the gravitational acceleration (9.81 m/s^2).

$$\phi_{x,y,t} = e^{i\omega t}\phi_1(x,y) \tag{S2}$$

Equation S2 into Equation S1 was shown in Equation S3.

$$\bigtriangledown \cdot (h \bigtriangledown \phi_1) e^{i\omega t} = -\frac{\omega^2}{g} e^{i\omega t} \tag{S3}$$

Dropping the subscript from ϕ , dividing by $e^{i\omega t}$, and $-\omega^2/g = \lambda$, then, the Equation S3. can be presented by the eigenvalue problem.

$$\bigtriangledown \cdot (h \bigtriangledown \phi) = \lambda\phi \tag{S4}$$

The differential form of the Equation S4 based on the bathymetries can be written as follows.

$$\frac{\partial}{\partial x}\left(h\frac{\partial\phi}{\partial x}\right) + \frac{\partial}{\partial y}\left(h\frac{\partial\phi}{\partial y}\right) = \lambda\phi \tag{S5}$$

The Equation S5 was modified to spherical coordinates in this study, as shown by Equation 2 in the main text and Equation S6:

$$\frac{1}{R^2 \cos\theta}\frac{\partial}{\partial\theta}\left(h\cos\theta\frac{\partial\phi}{\partial\theta}\right) + \frac{1}{R^2\cos\theta}\frac{\partial}{\partial\varphi}\left(h\frac{\partial\phi}{\partial\varphi}\right) = \lambda\phi \tag{S6}$$

where θ is the latitude, ϕ is the longitude, R is the Earth's radius. The Equation S6 was mapped to the grid in Figure S8 as follows.

$$\begin{aligned}\frac{1}{R^2\cos\theta\Delta\theta}&\left[h_{i+1/2,j}\cos(\theta+\Delta\theta)\frac{\phi_{i+1,j}-\phi_{i,j}}{\Delta\theta} - h_{i-1/2,j}\cos(\theta-\Delta\theta)\frac{\phi_{i,j}-\phi_{i-1,j}}{\Delta\theta}\right]\\ &+\frac{1}{R^2\cos\theta\Delta\varphi}\left[h_{i,j+1/2}\frac{\phi_{i,j+1}-\phi_{i,j}}{\Delta\varphi} - h_{i,j-1/2}\frac{\phi_{i,j}-\phi_{i,j-1}}{\Delta\varphi}\right] = \lambda\phi_{i,j}\end{aligned} \tag{S7}$$

The Equation S7 can be changed to the staggered grid system in the discrete form as follows.

$$\begin{aligned}\frac{h_{i+1/2,j}\cos(\theta+\Delta\theta)}{R^2\cos\theta\Delta\theta^2}\phi_{i+1,j} &+ \frac{h_{i-1/2,j}\cos(\theta-\Delta\theta)}{R^2\cos\theta\Delta\theta^2}\phi_{i-1,j} + \frac{h_{i,j+1/2}}{R^2\cos\theta\Delta\varphi^2}\phi_{i,j+1} + \frac{h_{i,j-1/2}}{R^2\cos\theta\Delta\varphi^2}\phi_{i,j-1}\\ &- \left[\frac{\cos(\theta+\Delta\theta)+\cos(\theta-\Delta\theta)}{R^2\cos\theta\Delta\theta^2}\left(h_{i+1/2,j}+h_{i-1/2,j}\right) + \frac{1}{R^2\cos\theta\Delta\varphi^2}\left(h_{i,j+1/2}+h_{i,j-1/2}\right)\right]\phi_{i,j}\\ &= \lambda\phi_{ij}\end{aligned} \tag{S8}$$

The Equation S8 can be presented in the simple form as follows.

$$a_{i+1,j}\phi_{i+1,j} + a_{i-1,j}\phi_{i-1,j} + a_{i,j+1}\phi_{i,j+1} + a_{i,j-1}\phi_{i,j-1} - a_{i,j}\phi_{i,j} = \lambda\phi_{ij} \tag{S9}$$

The terms $A_{i,j}$, $\phi_{i,j}$, and λ_i describe the seafloor, water level, and frequencies in the modal model. Assembly of Equation S10 at all grid gives rise to a matrix in the form as follows.

$$\vec{A}\cdot\vec{\phi} = \lambda\cdot\vec{\phi} \tag{S10}$$

The constructed matrix $\vec{A}$ is symmetric based on the number of the consideration grid for all the eigenvalues, as shown in its structure in Figure S9. The frequencies of the Modal analysis are given as follows.

$$f_i = \omega_i/2\pi = \sqrt{-g\lambda_i}/2\pi, i = 1,2,...,n \tag{S11}$$

where n is the number of the consideration grid.

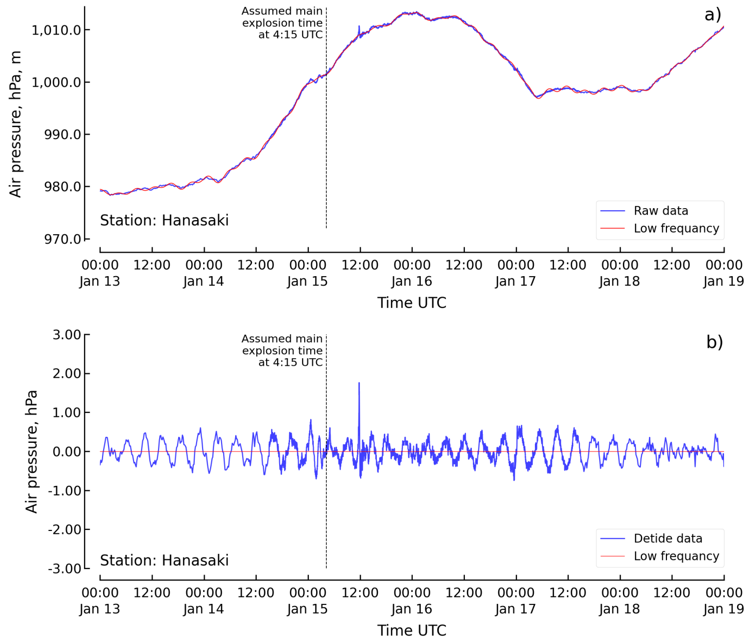

Fig. S1. Atmospheric pressure waveform from the **Hanasaki** observation point. a) Raw waveform data and low-frequency trend (from the high-pass filter), b) Filtered waveform data from the high-pass filter.

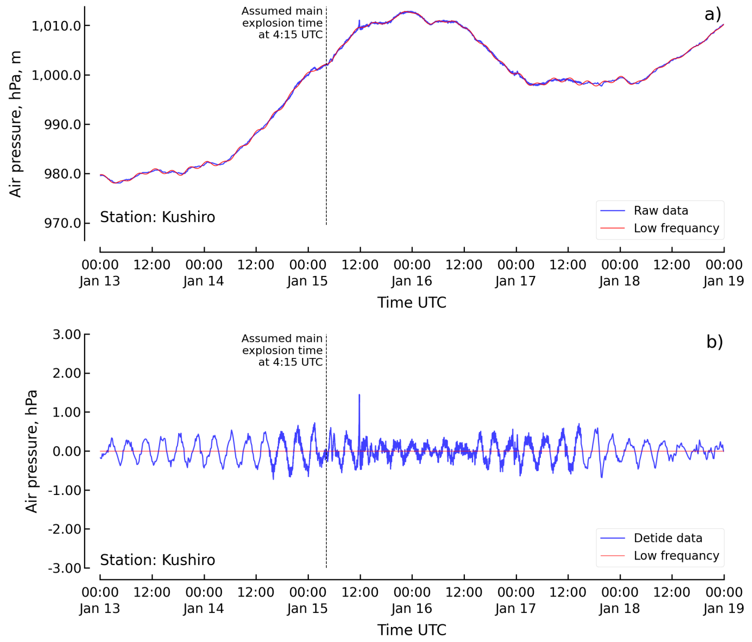

Fig. S1 (Cont.). Atmospheric pressure waveform from the **Kushiro** observation point. a) Raw waveform data and low-frequency trend (from the high-pass filter), b) Filtered waveform data from the high-pass filter.

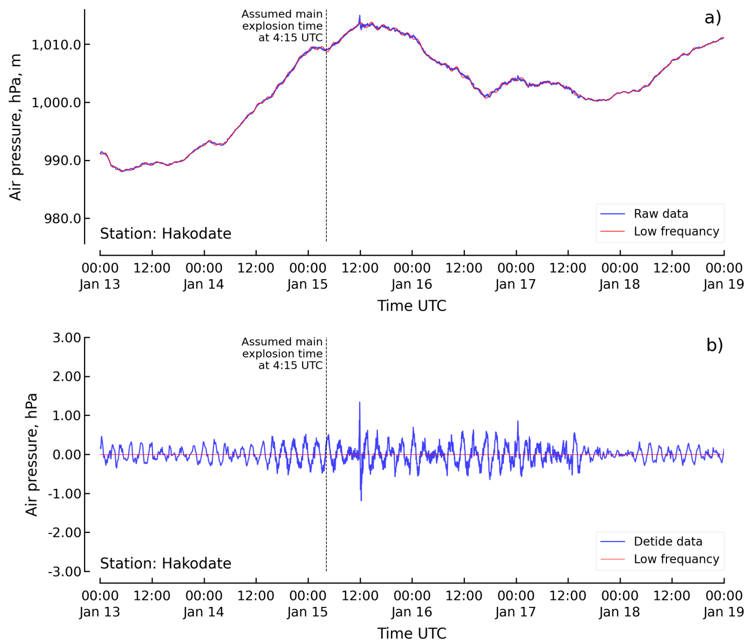

Fig. S1 (Cont.). Atmospheric pressure waveform from the **Hakodate** observation point. a) Raw waveform data and low-frequency trend (from the high-pass filter), b) Filtered waveform data from the high-pass filter.

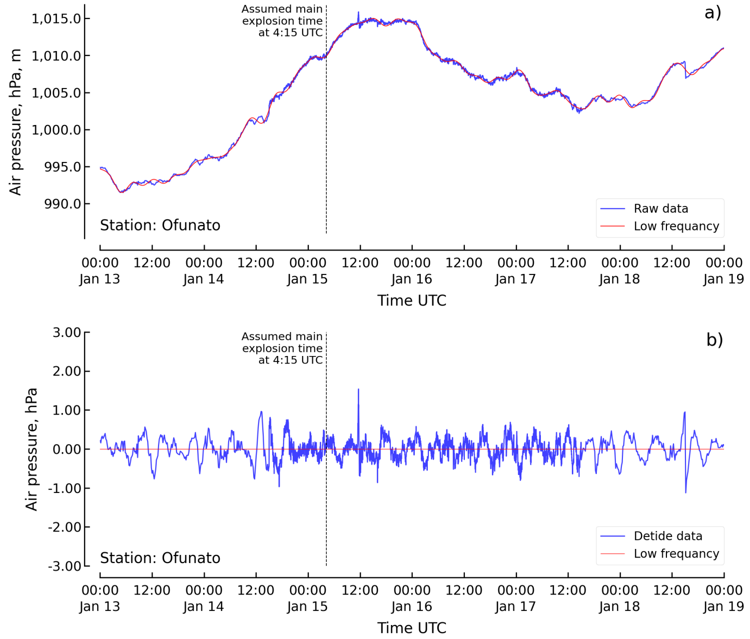

Fig. S1 (Cont.). Atmospheric pressure waveform from the **Ofunato** observation point. a) Raw waveform data and low-frequency trend (from the high-pass filter), b) Filtered waveform data from the high-pass filter.

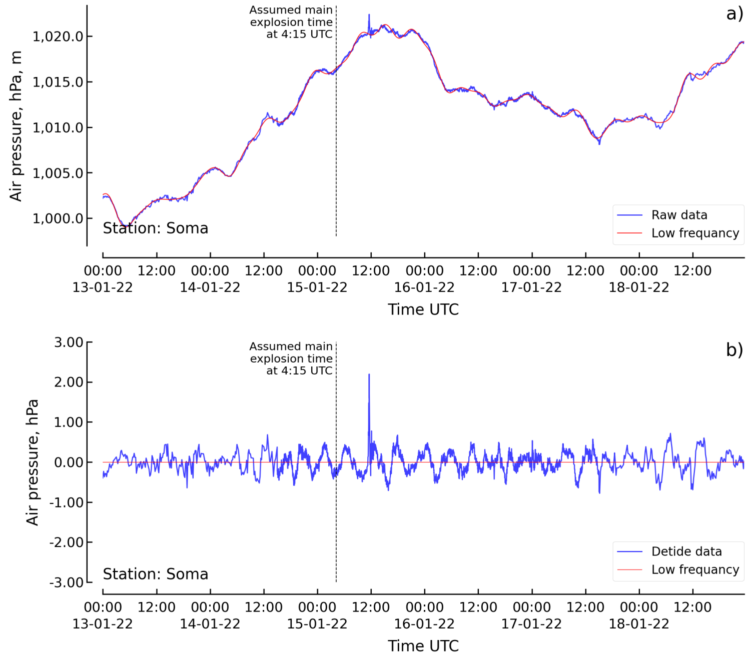

Fig. S1 (Cont.). Atmospheric pressure waveform from the **Soma** observation point. a) Raw waveform data and low-frequency trend (from the high-pass filter), b) Filtered waveform data from the high-pass filter.

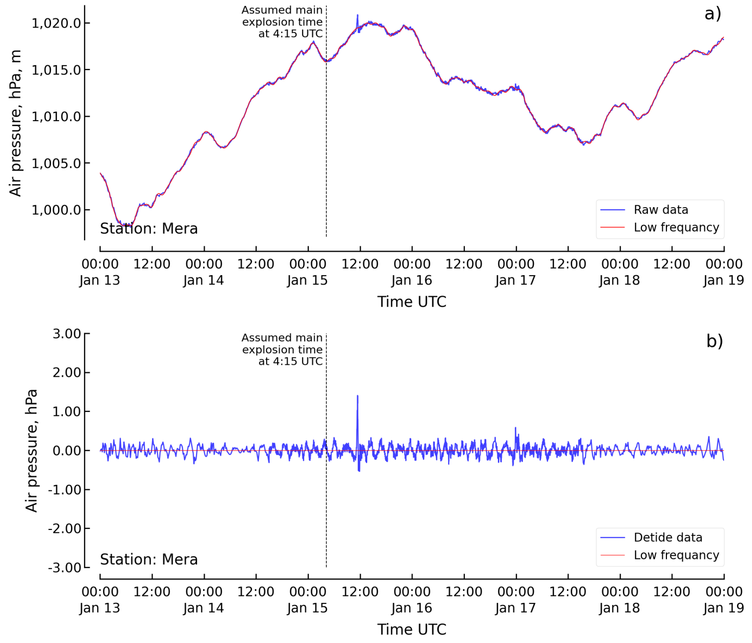

Fig. S1 (Cont.). Atmospheric pressure waveform from the **Mera** observation point. a) Raw waveform data and low-frequency trend (from the high-pass filter), b) Filtered waveform data from the high-pass filter.

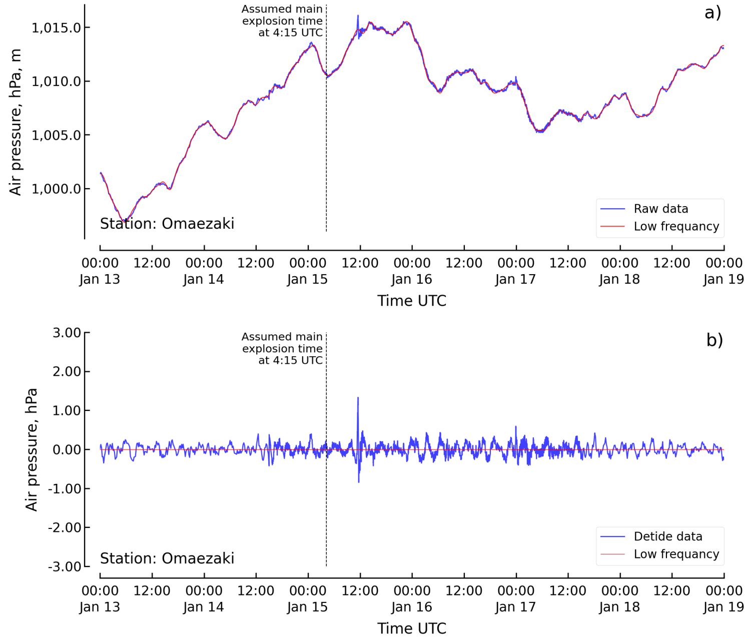

Fig. S1 (Cont.). Atmospheric pressure waveform from the **Omaezaki** observation point. a) Raw waveform data and low-frequency trend (from the high-pass filter), b) Filtered waveform data from the high-pass filter.

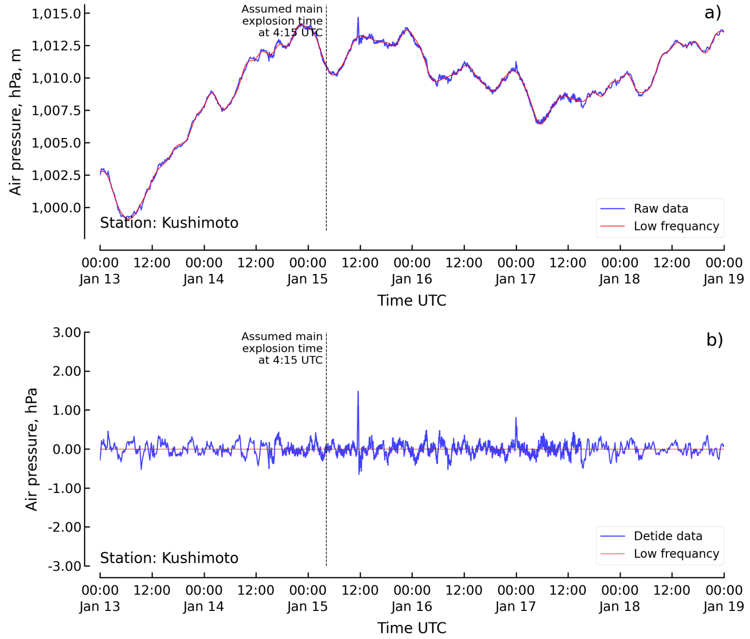

Fig. S1 (Cont.). Atmospheric pressure waveform from the **Kushimoto** observation point. a) Raw waveform data and low-frequency trend (from the high-pass filter), b) Filtered waveform data from the high-pass filter.

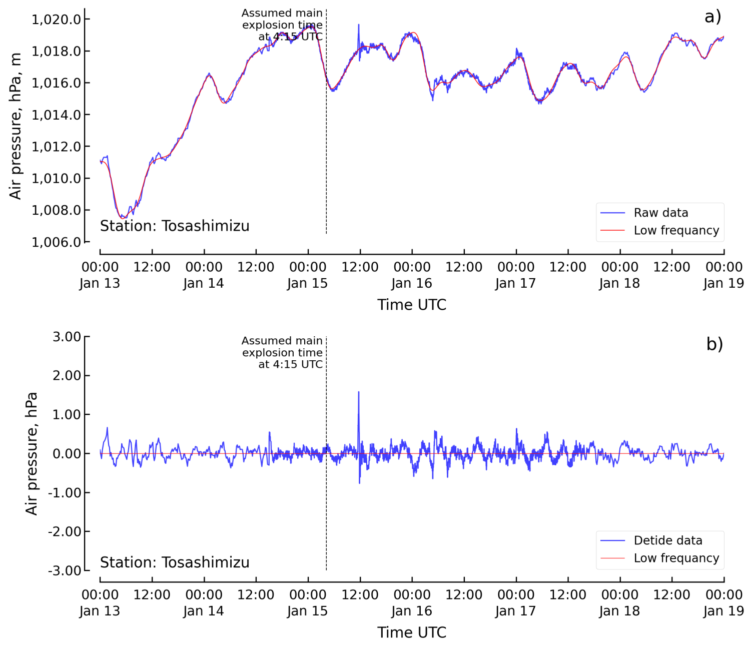

Fig. S1 (Cont.). Atmospheric pressure waveform from the **Tosashimizu** observation point. a) Raw waveform data and low-frequency trend (from the high-pass filter), b) Filtered waveform data from the high-pass filter.

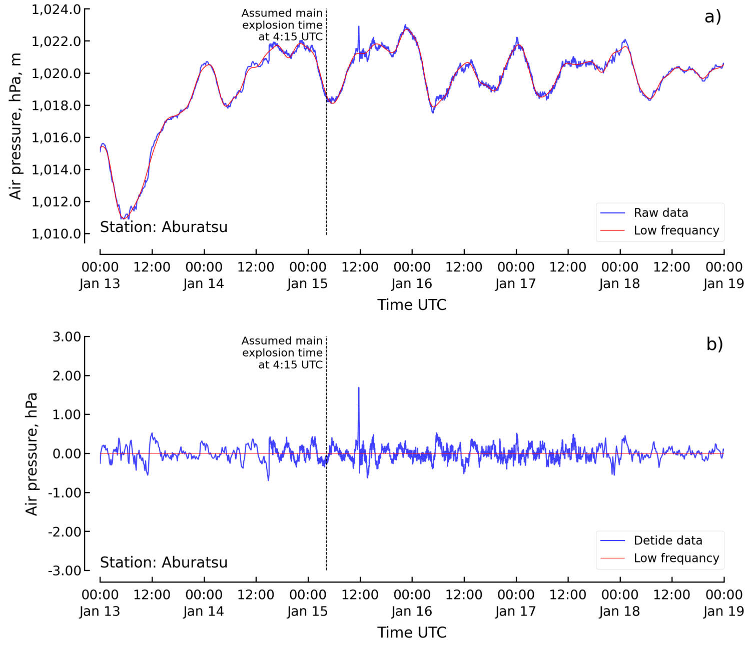

Fig. S1 (Cont.). Atmospheric pressure waveform from the **Aburatsu** observation point. a) Raw waveform data and low-frequency trend (from the high-pass filter), b) Filtered waveform data from the high-pass filter.

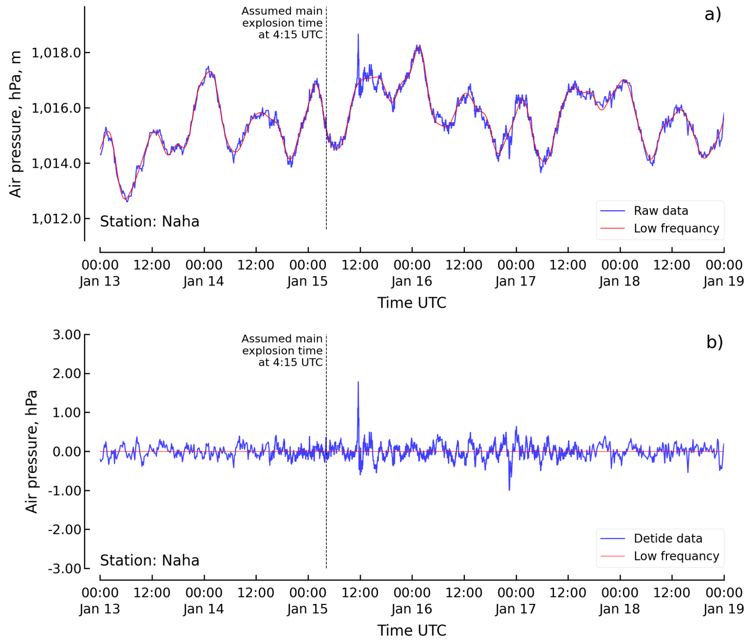

Fig. S1 (Cont.). Atmospheric pressure waveform from the **Naha** observation point. a) Raw waveform data and low-frequency trend (from the high-pass filter), b) Filtered waveform data from the high-pass filter.

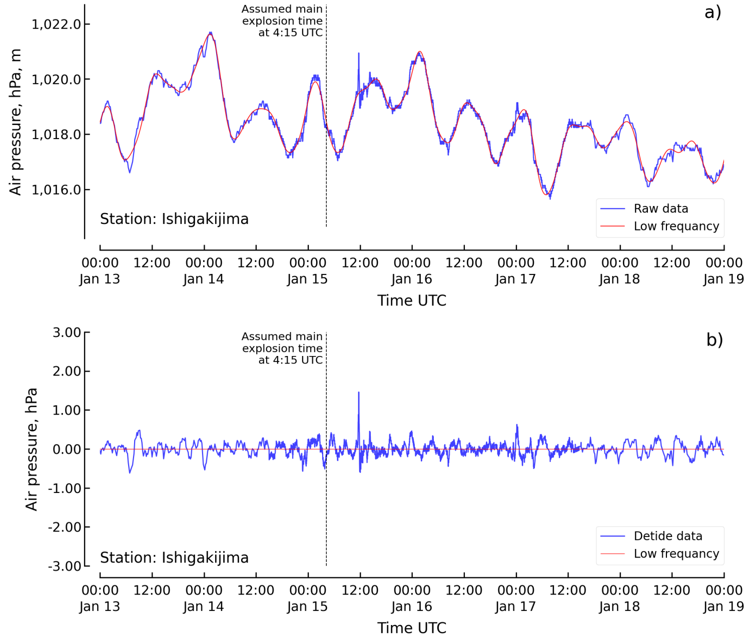

Fig. S1 (Cont.). Atmospheric pressure waveform from the **Ishigakijima** observation point. a) Raw waveform data and low-frequency trend (from the high-pass filter), b) Filtered waveform data from the high-pass filter.

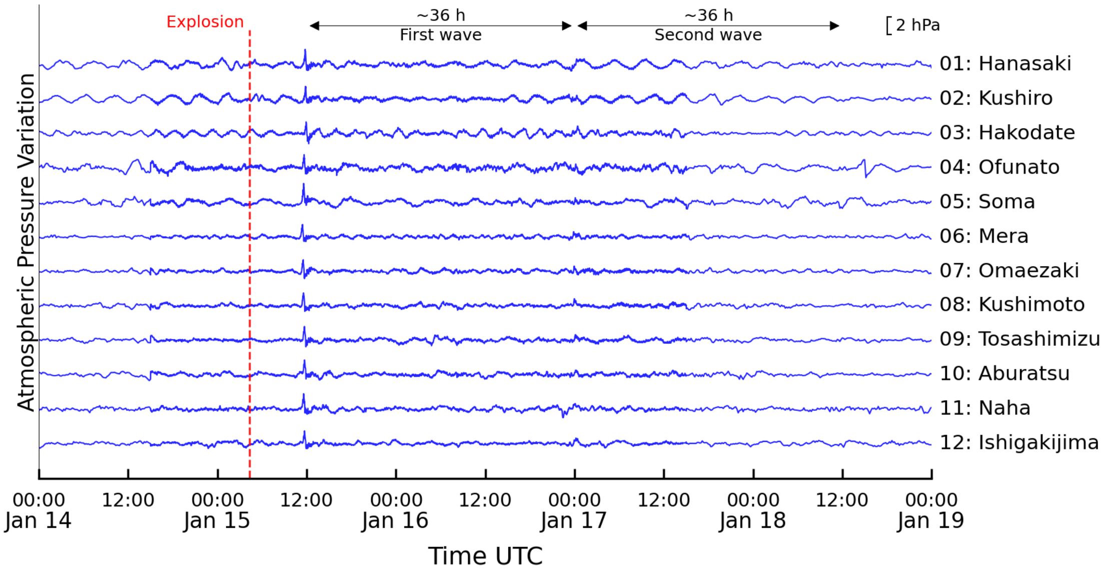

Fig. S2. Time series of atmospheric pressure records from the 12 stations. The largest explosion from HTHH occurred on 04:14:45 UTC, 15 January 2022, and is marked with a red dashed line. The first (12:00 UTC, 15 January 2022 to 00:00 UTC, 17 January 2022) and second (00:00 UTC, 17 January 2022 to 12:00 UTC, 18 January 2022) wave components were approximately 36 hours.

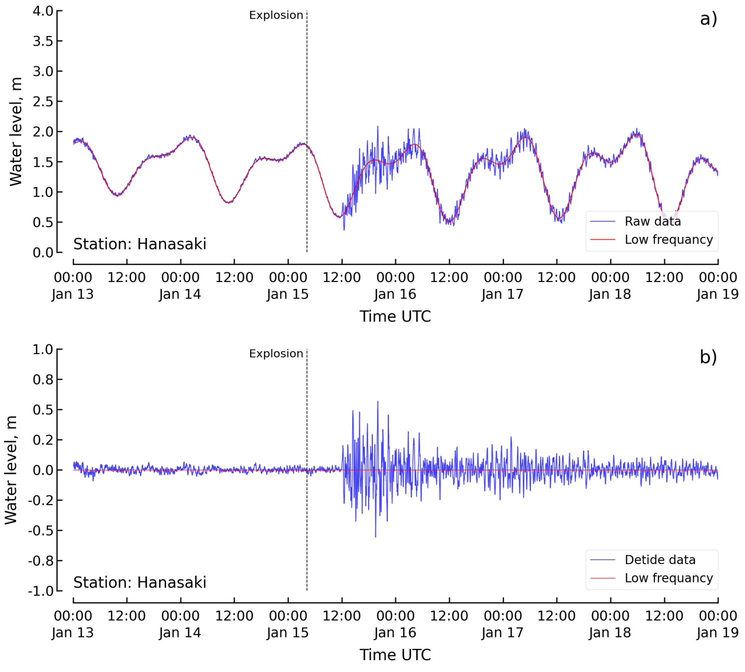

Fig. S3. The tsunami waveform from the **Hanasaki** observation point. a) Raw waveform data and low-frequency trend (from the high-pass filter), b) Filtered waveform data from the high-pass filter.

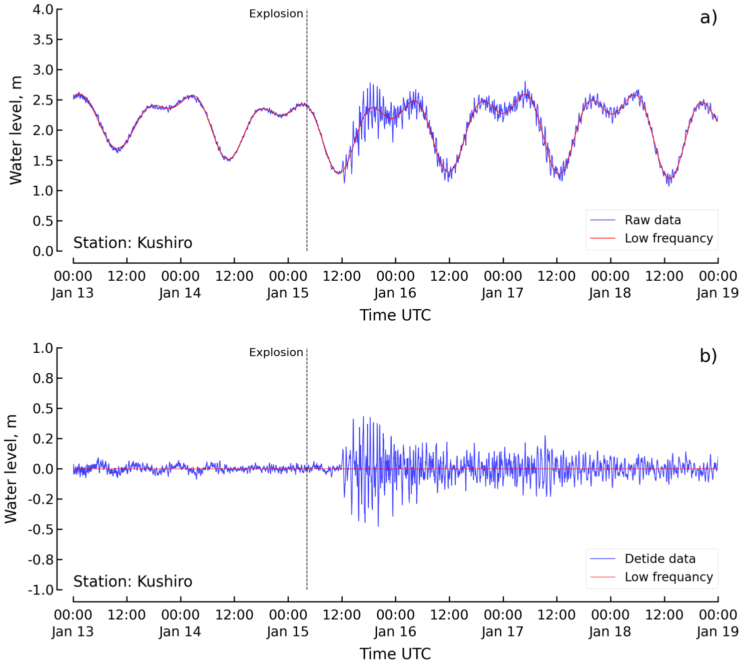

Fig. S3 (Cont.). The tsunami waveform from the **Kushiro** observation point. a) Raw waveform data and low-frequency trend (from the high-pass filter), b) Filtered waveform data from the high-pass filter.

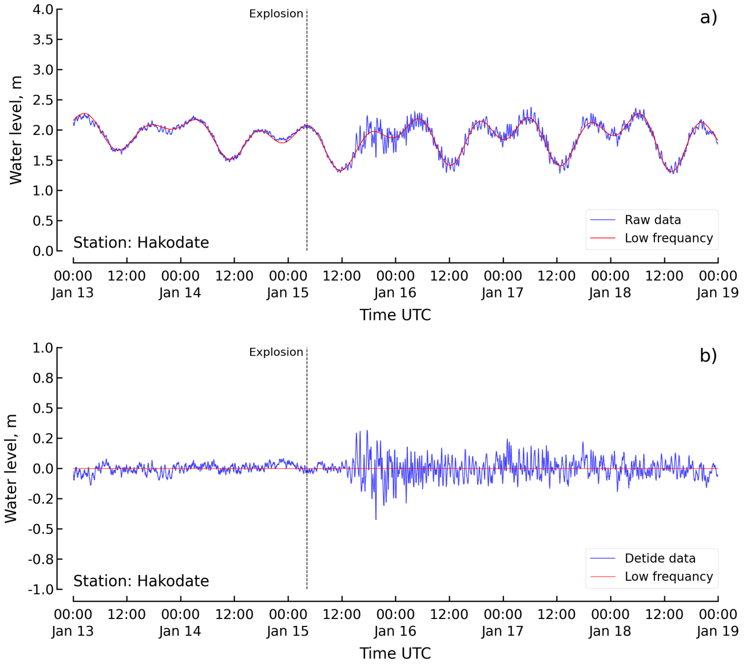

Fig. S3 (Cont.). The tsunami waveform from the **Hakodate** observation point. a) Raw waveform data and low-frequency trend (from the high-pass filter), b) Filtered waveform data from the high-pass filter.

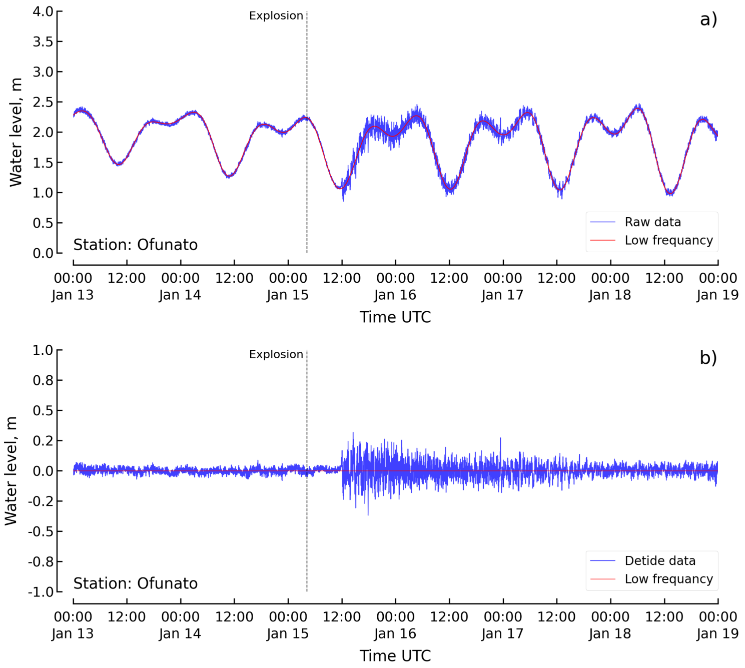

Fig. S3 (Cont.). The tsunami waveform from the **Ofunato** observation point. a) Raw waveform data and low-frequency trend (from the high-pass filter), b) Filtered waveform data from the high-pass filter.

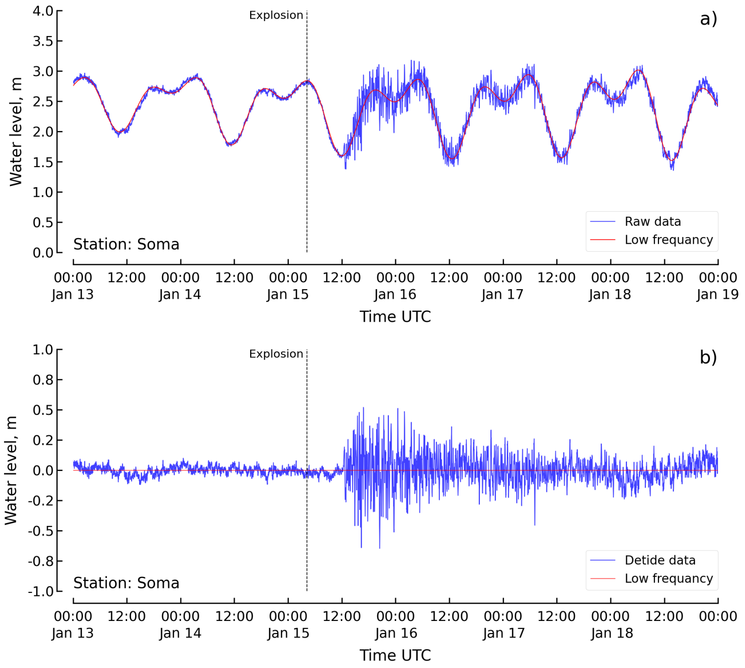

Fig. S3 (Cont.). The tsunami waveform from the **Soma** observation point. a) Raw waveform data and low-frequency trend (from the high-pass filter), b) Filtered waveform data from the high-pass filter.

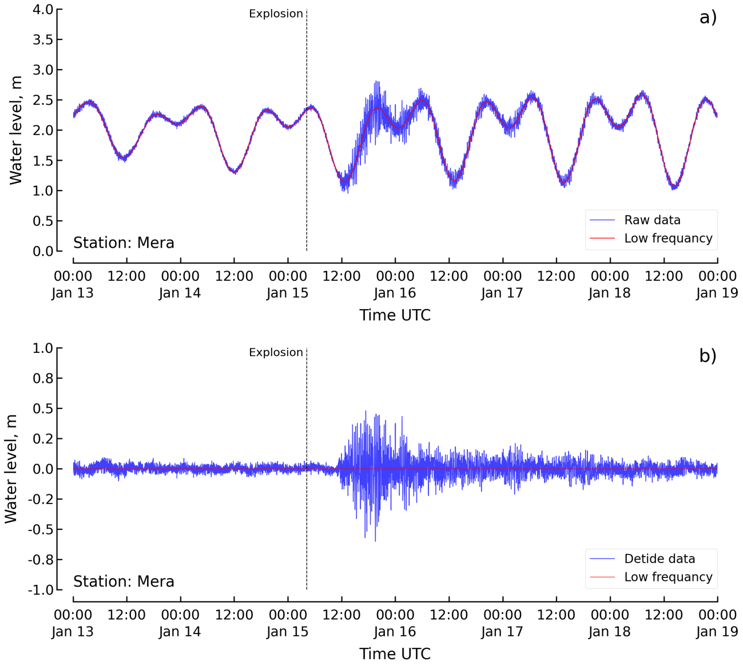

Fig. S3 (Cont.). The tsunami waveform from the **Mera** observation point. a) Raw waveform data and low-frequency trend (from the high-pass filter), b) Filtered waveform data from the high-pass filter.

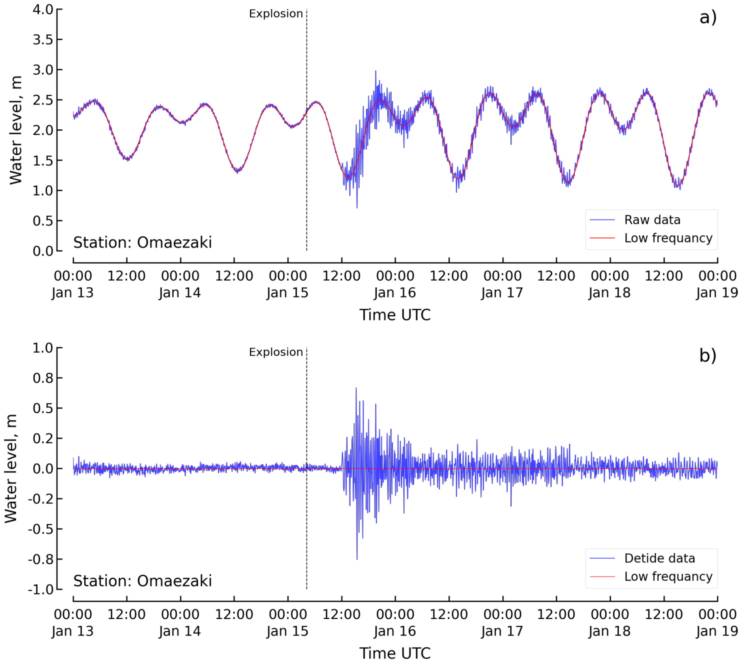

Fig. S3 (Cont.). The tsunami waveform from the **Omaezaki** observation point. a) Raw waveform data and low-frequency trend (from the high-pass filter), b) Filtered waveform data from the high-pass filter.

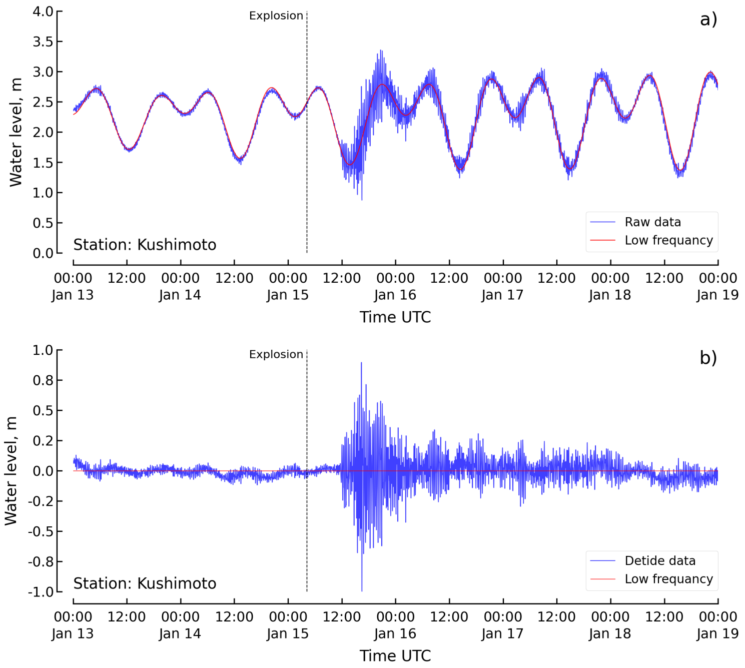

Fig. S3 (Cont.). The tsunami waveform from the **Kushimoto** observation point. a) Raw waveform data and low-frequency trend (from the high-pass filter), b) Filtered waveform data from the high-pass filter.

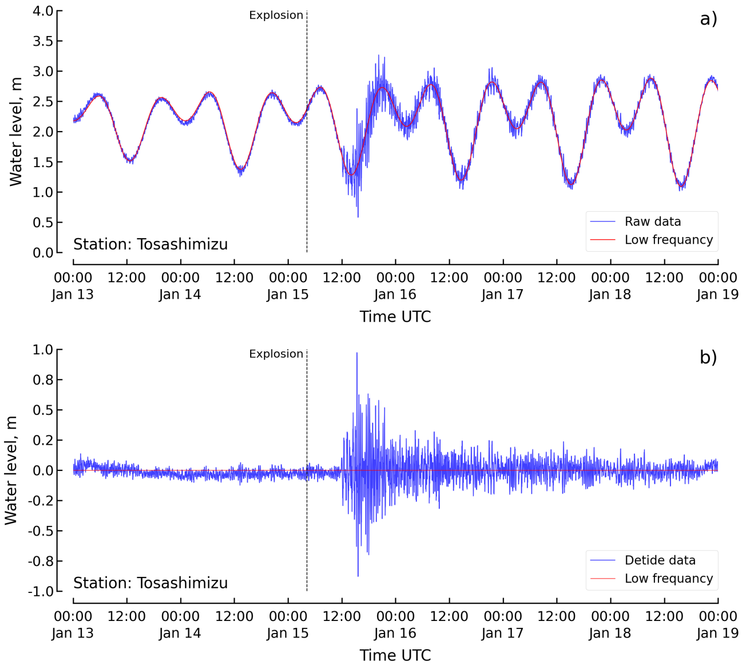

Fig. S3 (Cont.). The tsunami waveform from the **Tosashimizu** observation point. a) Raw waveform data and low-frequency trend (from the high-pass filter), b) Filtered waveform data from the high-pass filter.

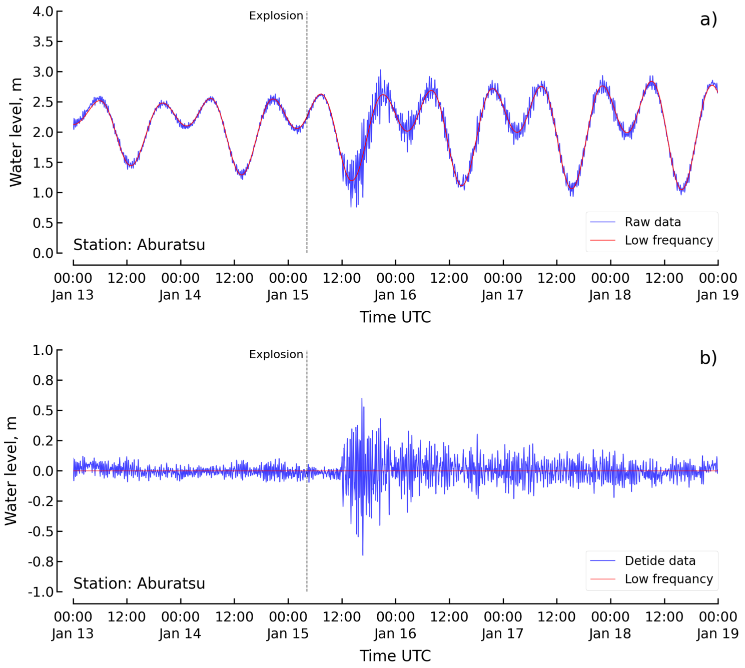

Fig. S3 (Cont.). The tsunami waveform from the **Aburatsu** observation point. a) Raw waveform data and low-frequency trend (from the high-pass filter), b) Filtered waveform data from the high-pass filter.

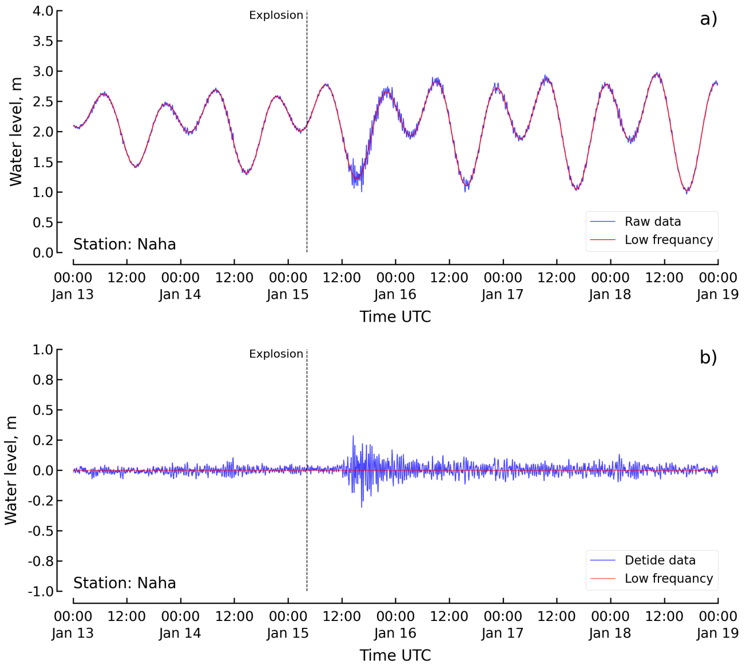

Fig. S3 (Cont.). The tsunami waveform from the **Naha** observation point. a) Raw waveform data and low-frequency trend (from the high-pass filter), b) Filtered waveform data from the high-pass filter.

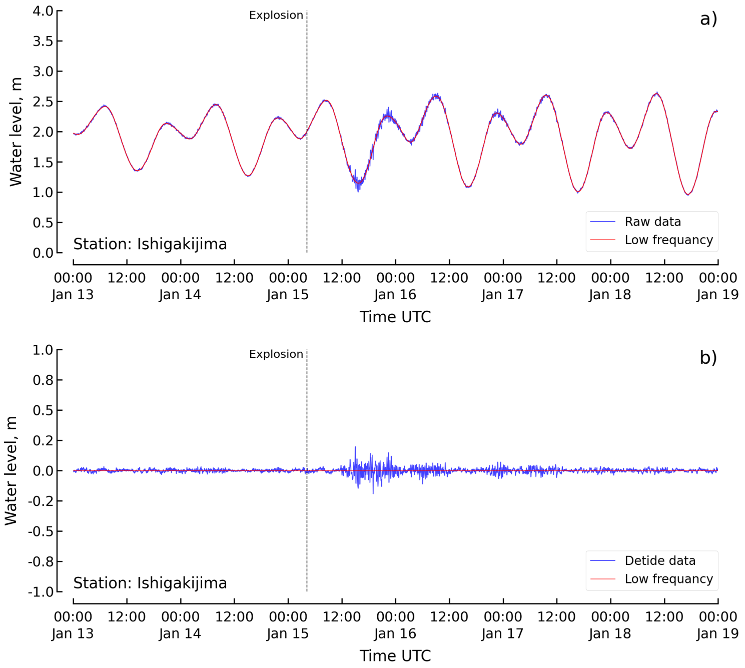

Fig. S3 (Cont.). The tsunami waveform from the **Ishigakijima** observation point. a) Raw waveform data and low-frequency trend (from the high-pass filter), b) Filtered waveform data from the high-pass filter.

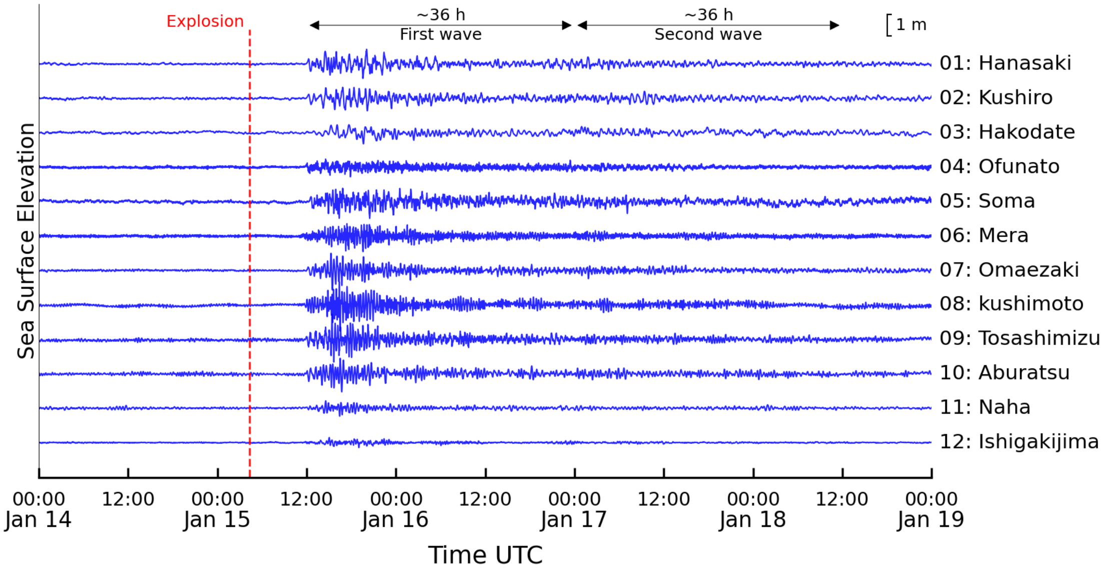

Fig. S4. Time series of sea surface elevation records from the 12 stations. The largest explosion of HTHH occurred on 04:14:45 UTC, 15 January 2022, and is indicated by a red dashed line. The first (12:00 UTC, 15 January 2022 to 00:00 UTC, 17 January 2022) and second (00:00 UTC, 17 January 2022 to 12:00 UTC, 18 January 2022) wave components were separated by approximately 36 hours.

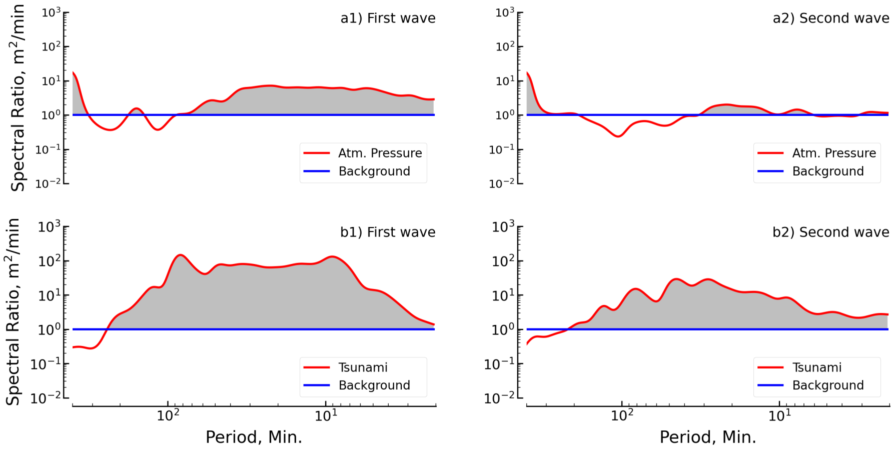

Fig. S5. Spectra ratios of the atmospheric pressure wave and tsunami wave for the **Hanasaki** station. Red lines are the ratio between the wave spectra and background spectra. The top row presents the spectral ratio of the atmospheric pressure, and the bottom row presents the spectral ratio of the tsunami wave. The first column presents the spectral ratio of the first wave, and the second column presents the spectral ratio of the second wave.

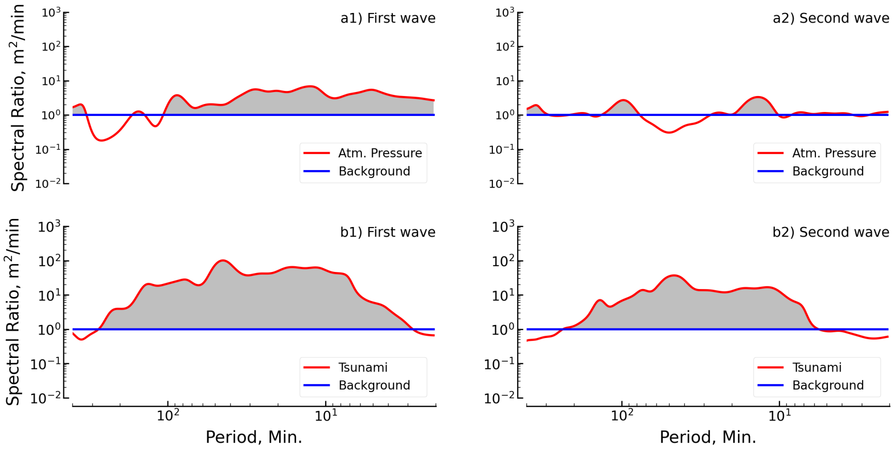

Fig. S5 (Cont.). Spectra ratios of the atmospheric pressure wave and tsunami wave for the **Kushiro** station. Red lines are the ratio between the wave spectra and background spectra. The top row presents the spectral ratio of the atmospheric pressure, and the bottom row presents the spectral ratio of the tsunami wave. The first column presents the spectral ratio of the first wave, and the second column presents the spectral ratio of the second wave.

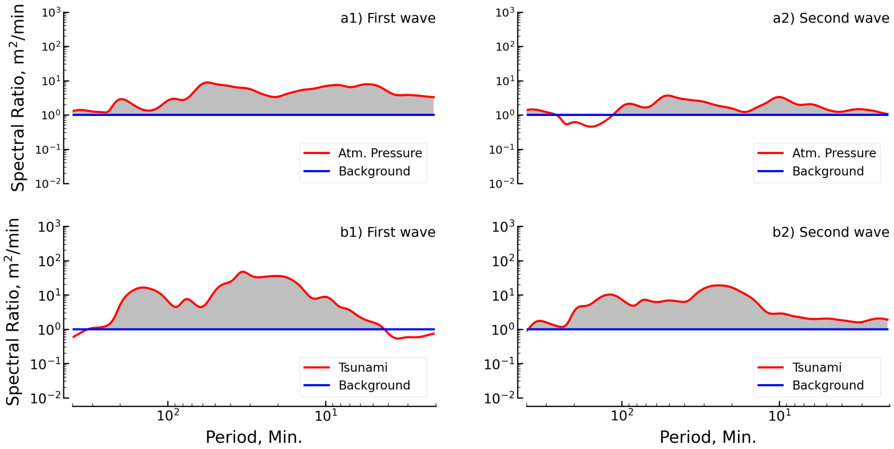

Fig. S5 (Cont.). Spectra ratios of the atmospheric pressure wave and tsunami wave for the **Hakodatei** station. Red lines are the ratio between the wave spectra and background spectra. The top row presents the spectral ratio of the atmospheric pressure, and the bottom row presents the spectral ratio of the tsunami wave. The first column presents the spectral ratio of the first wave, and the second column presents the spectral ratio of the second wave.

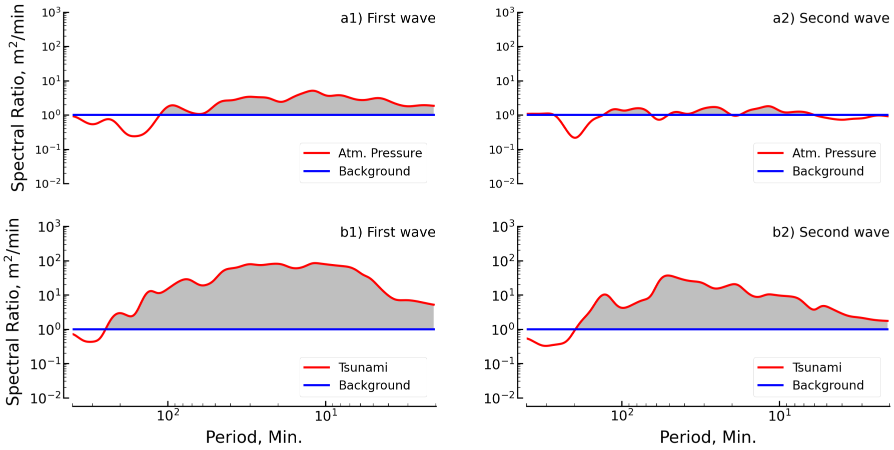

Fig. S5 (Cont.). Spectra ratios of the atmospheric pressure wave and tsunami wave for the **Ofunato** station. Red lines are the ratio between the wave spectra and background spectra. The top row presents the spectral ratio of the atmospheric pressure, and the bottom row presents the spectral ratio of the tsunami wave. The first column presents the spectral ratio of the first wave, and the second column presents the spectral ratio of the second wave.

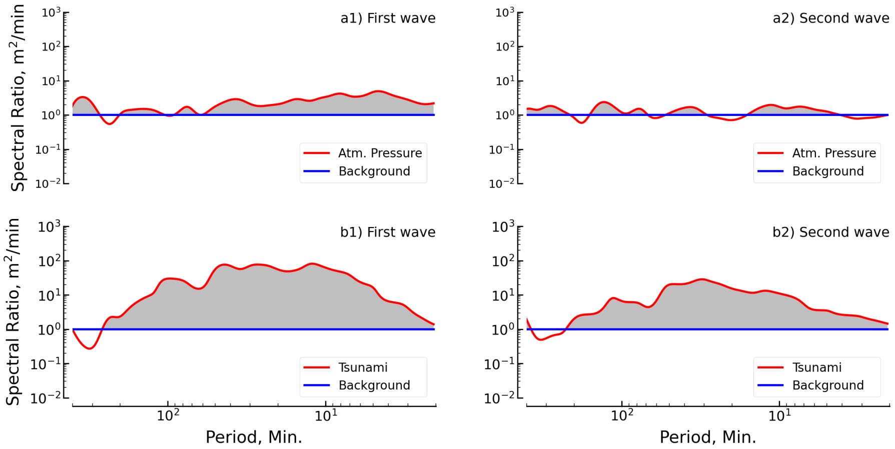

Fig. S5 (Cont.). Spectra ratios of the atmospheric pressure wave and tsunami wave for the **Soma** station. Red lines are the ratio between the wave spectra and background spectra. The top row presents the spectral ratio of the atmospheric pressure, and the bottom row presents the spectral ratio of the tsunami wave. The first column presents the spectral ratio of the first wave, and the second column presents the spectral ratio of the second wave.

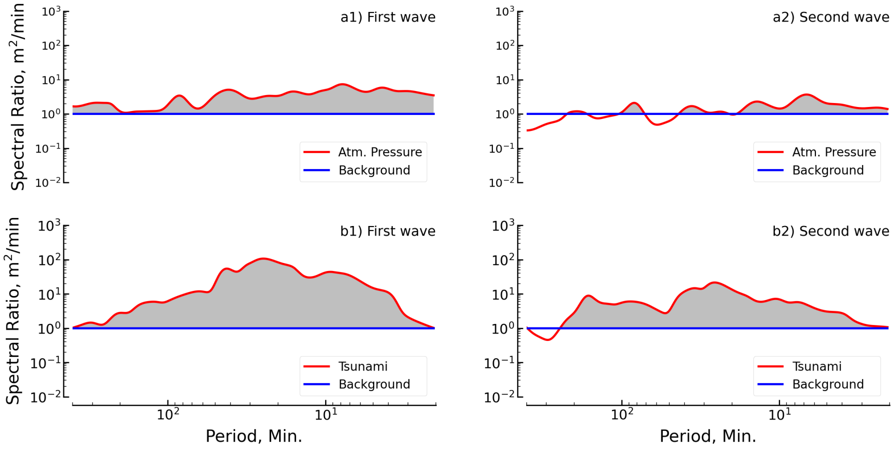

Fig. S5 (Cont.). Spectra ratios of the atmospheric pressure wave and tsunami wave for the **Mera** station. Red lines are the ratio between the wave spectra and background spectra. The top row presents the spectral ratio of the atmospheric pressure, and the bottom row presents the spectral ratio of the tsunami wave. The first column presents the spectral ratio of the first wave, and the second column presents the spectral ratio of the second wave.

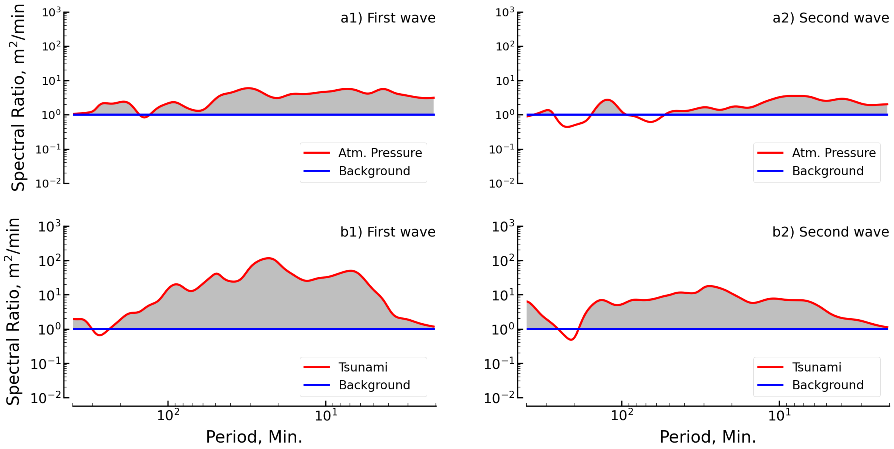

Fig. S5 (Cont.). Spectra ratios of the atmospheric pressure wave and tsunami wave for the **Omaezaki** station. Red lines are the ratio between the wave spectra and background spectra. The top row presents the spectral ratio of the atmospheric pressure, and the bottom row presents the spectral ratio of the tsunami wave. The first column presents the spectral ratio of the first wave, and the second column presents the spectral ratio of the second wave.

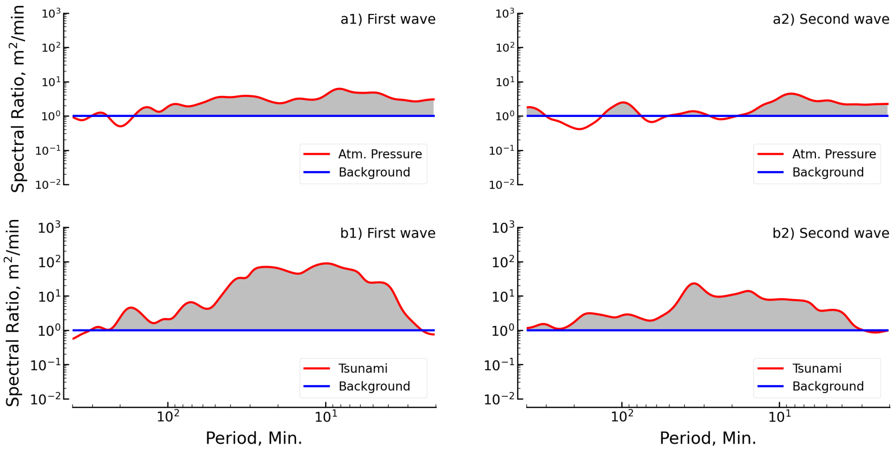

Fig. S5 (Cont.). Spectra ratios of the atmospheric pressure wave and tsunami wave for the **Kushimoto** station. Red lines are the ratio between the wave spectra and background spectra. The top row presents the spectral ratio of the atmospheric pressure, and the bottom row presents the spectral ratio of the tsunami wave. The first column presents the spectral ratio of the first wave, and the second column presents the spectral ratio of the second wave.

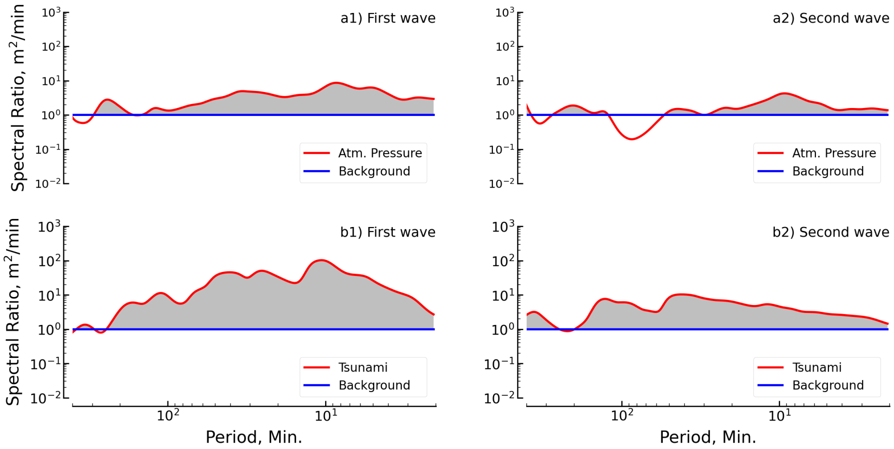

Fig. S5 (Cont.). Spectra ratios of the atmospheric pressure wave and tsunami wave for the **Tosashimizu** station. Red lines are the ratio between the wave spectra and background spectra. The top row presents the spectral ratio of the atmospheric pressure, and the bottom row presents the spectral ratio of the tsunami wave. The first column presents the spectral ratio of the first wave, and the second column presents the spectral ratio of the second wave.

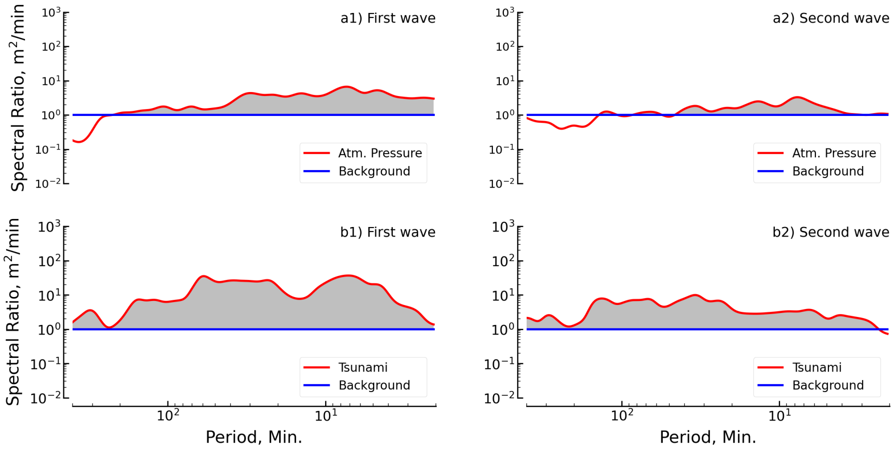

Fig. S5 (Cont.). Spectra ratios of the atmospheric pressure wave and tsunami wave for the **Aburatsu** station. Red lines are the ratio between the wave spectra and background spectra. The top row presents the spectral ratio of the atmospheric pressure, and the bottom row presents the spectral ratio of the tsunami wave. The first column presents the spectral ratio of the first wave, and the second column presents the spectral ratio of the second wave.

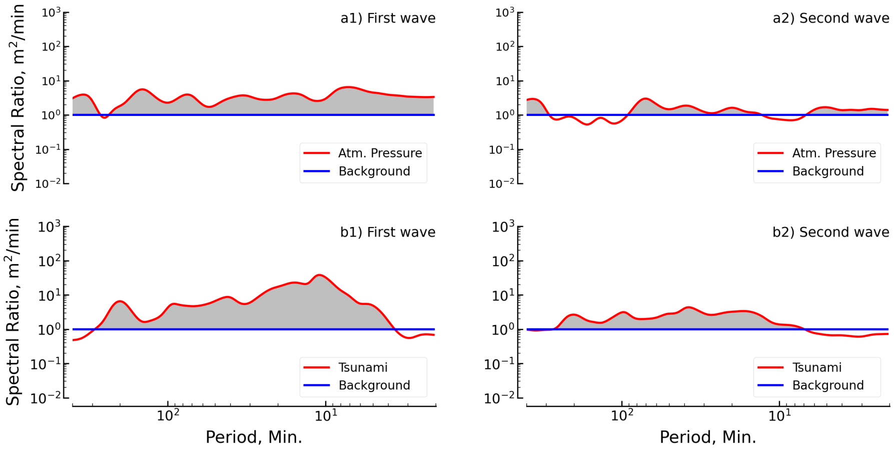

Fig. S5 (Cont.). Spectra ratios of the atmospheric pressure wave and tsunami wave for the **Naha** station. Red lines are the ratio between the wave spectra and background spectra. The top row presents the spectral ratio of the atmospheric pressure, and the bottom row presents the spectral ratio of the tsunami wave. The first column presents the spectral ratio of the first wave, and the second column presents the spectral ratio of the second wave.

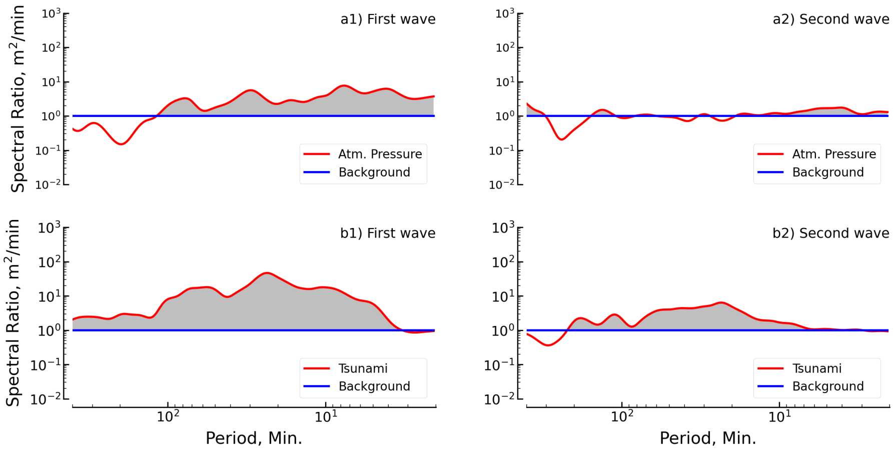

Fig. S5 (Cont.). Spectra ratios of the atmospheric pressure wave and tsunami wave for the **Ishigakijima** station. Red lines are the ratio between the wave spectra and background spectra. The top row presents the spectral ratio of the atmospheric pressure, and the bottom row presents the spectral ratio of the tsunami wave. The first column presents the spectral ratio of the first wave, and the second column presents the spectral ratio of the second wave.

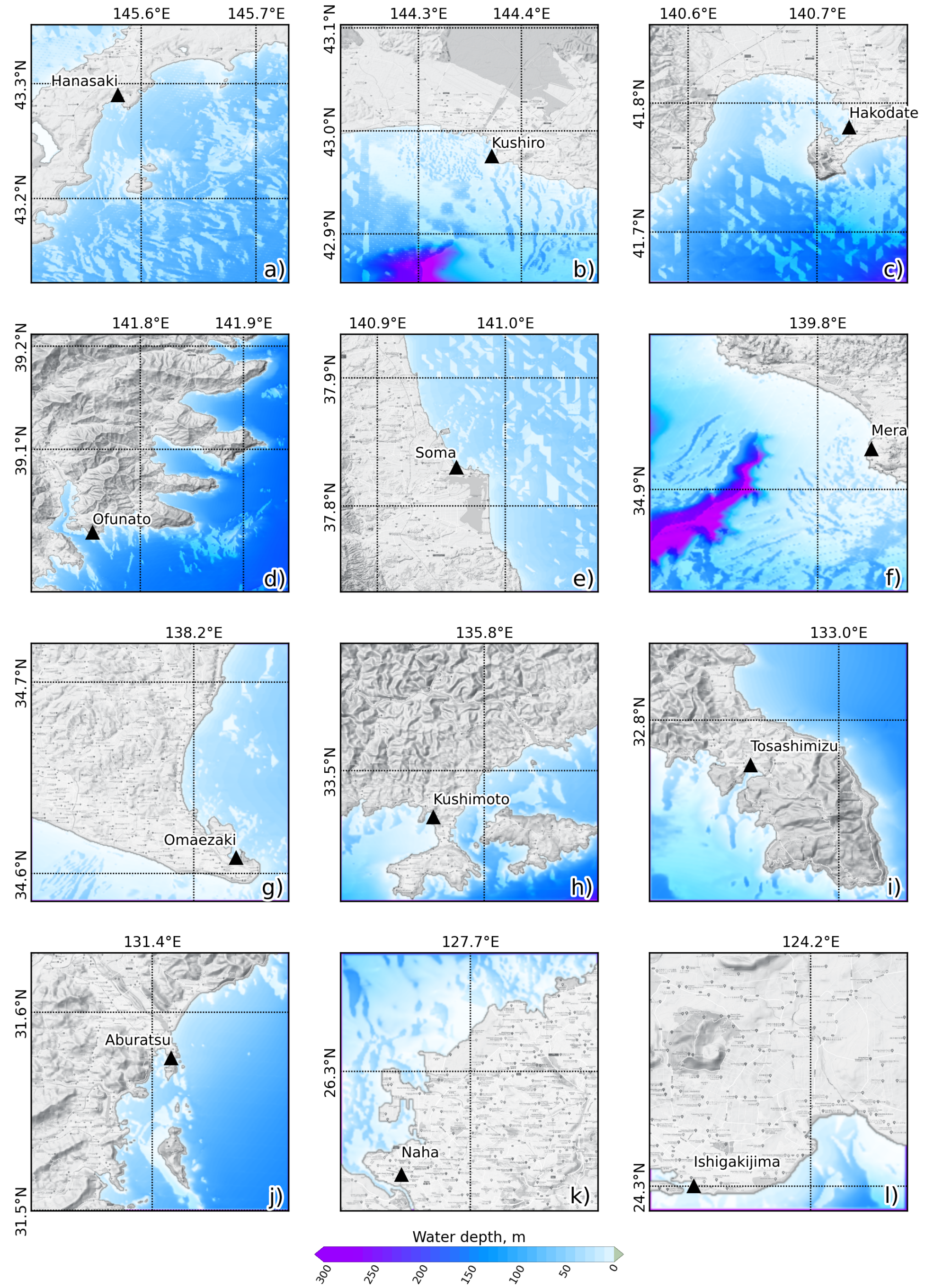

Fig. S6. Bathymetry data for the regional domain that covers the bay where the selected observation stations are located. Contours are in 10 m intervals. The maps were generated using Python version 3.8 (http://www.python.org) with the Matplotlib library (https://matplotlib.org/), and the basemap was downloaded from the QuickMapServices plugin (https://nextgis.com/blog/quickmapservices/) through QGIS (http://www.qgis.org).

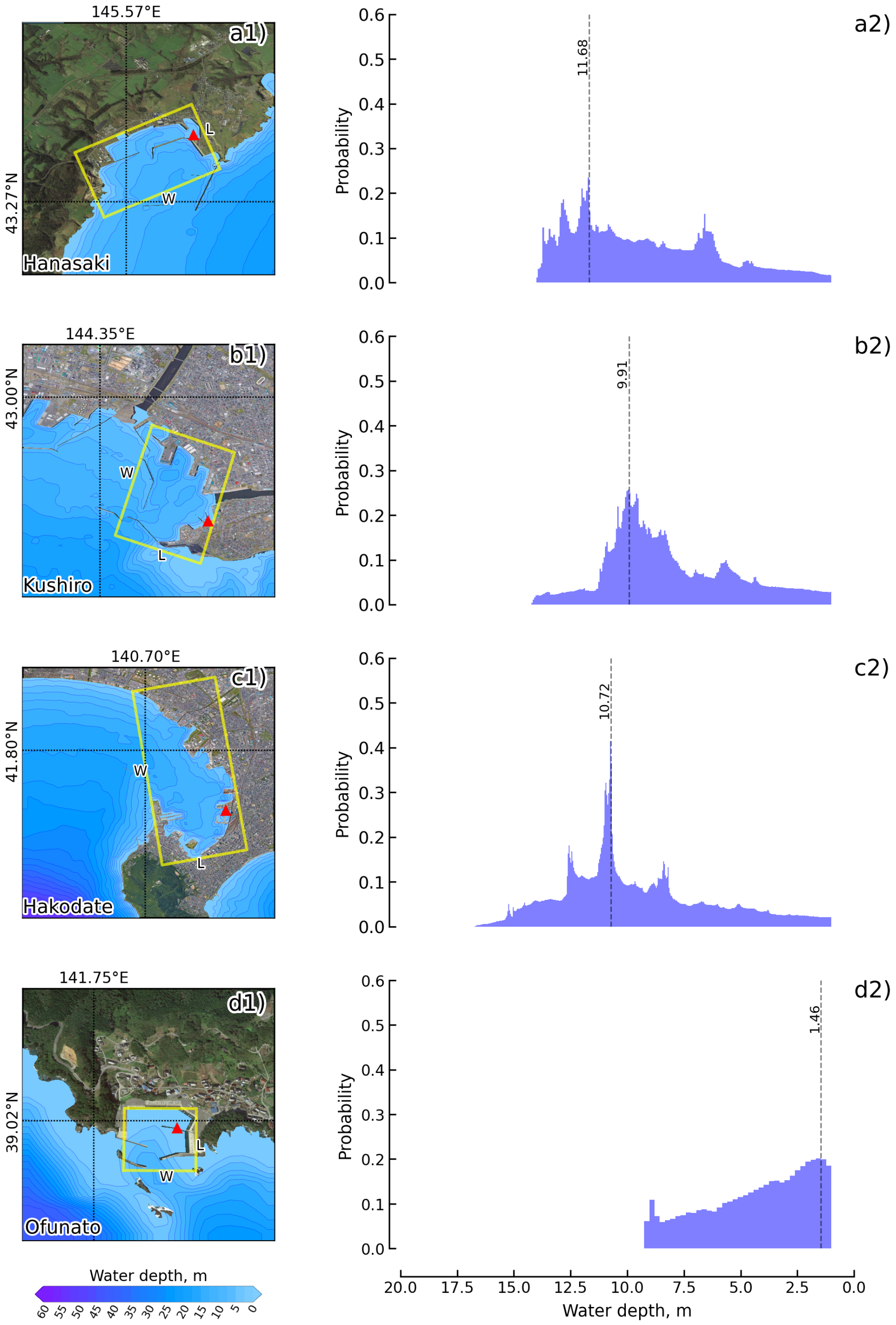

Fig. S7. Left panel) Bathymetry data for the regional domain that covers the bay where the selected observation stations are located. Contours are marked in 2.5 m intervals. The approximated geometry of the port is represented by the yellow rectangle, which was used to estimate the local oscillation modes. The maps were generated using Python version 3.8 (http://www.python.org) with the Matplotlib library (https://matplotlib.org/), and the basemap was downloaded from the QuickMapServices plugin (https://nextgis.com/blog/quickmapservices/) through QGIS (http://www.qgis.org). Right panel) A histogram of water depth in the port was used to identify the representative port depth to estimate the local oscillations.

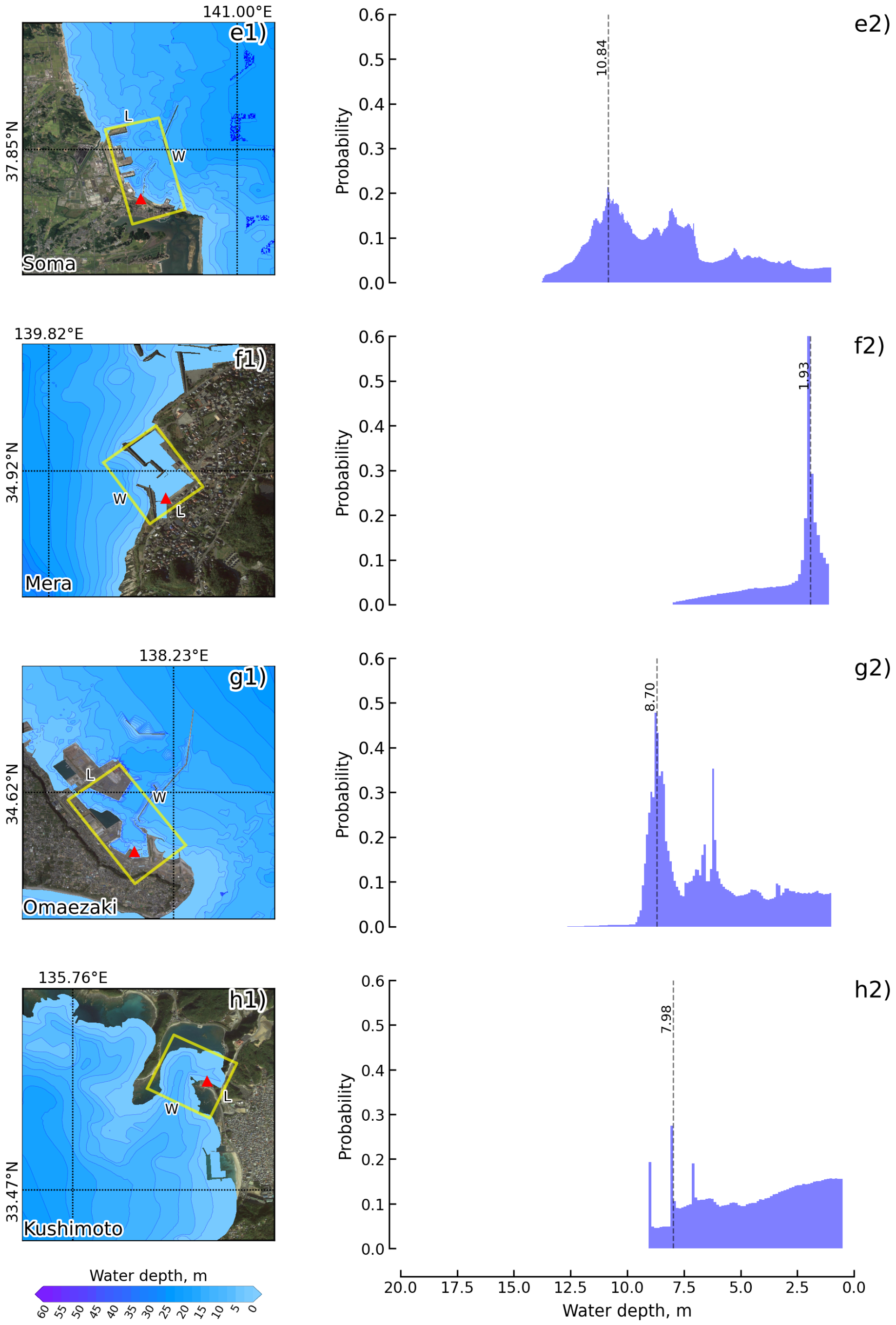

Fig. S7 (Cont.). Left panel) Bathymetry data for the regional domain that covers the bay where the selected observation stations are located. Contours are marked in 2.5 m intervals. The approximated geometry of the port is represented by the yellow rectangle, which was used to estimate the local oscillation modes. The maps were generated using Python version 3.8 (http://www.python.org) with the Matplotlib library (https://matplotlib.org/), and the basemap was downloaded from the QuickMapServices plugin (https://nextgis.com/blog/quickmapservices/) through QGIS (http://www.qgis.org). Right panel) A histogram of water depth in the port was used to identify the representative port depth to estimate the local oscillations.

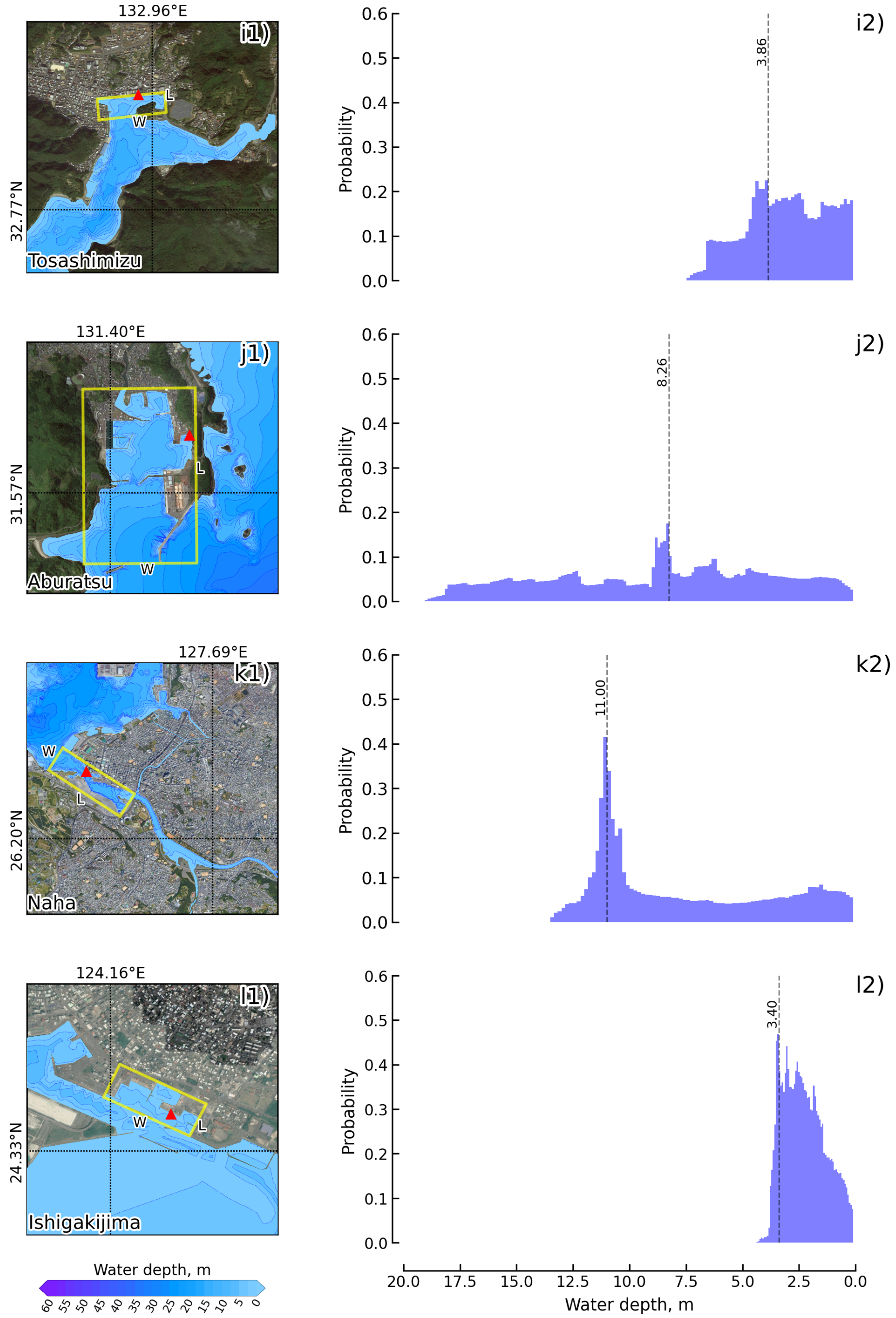

Fig. S7 (Cont.). Left panel) Bathymetry data for the regional domain that covers the bay where the selected observation stations are located. Contours are marked in 2.5 m intervals. The approximated geometry of the port is represented by the yellow rectangle, which was used to estimate the local oscillation modes. The maps were generated using Python version 3.8 (http://www.python.org) with the Matplotlib library (https://matplotlib.org/), and the basemap was downloaded from the QuickMapServices plugin (https://nextgis.com/blog/quickmapservices/) through QGIS (http://www.qgis.org). Right panel) A histogram of water depth in the port was used to identify the representative port depth to estimate the local oscillations.

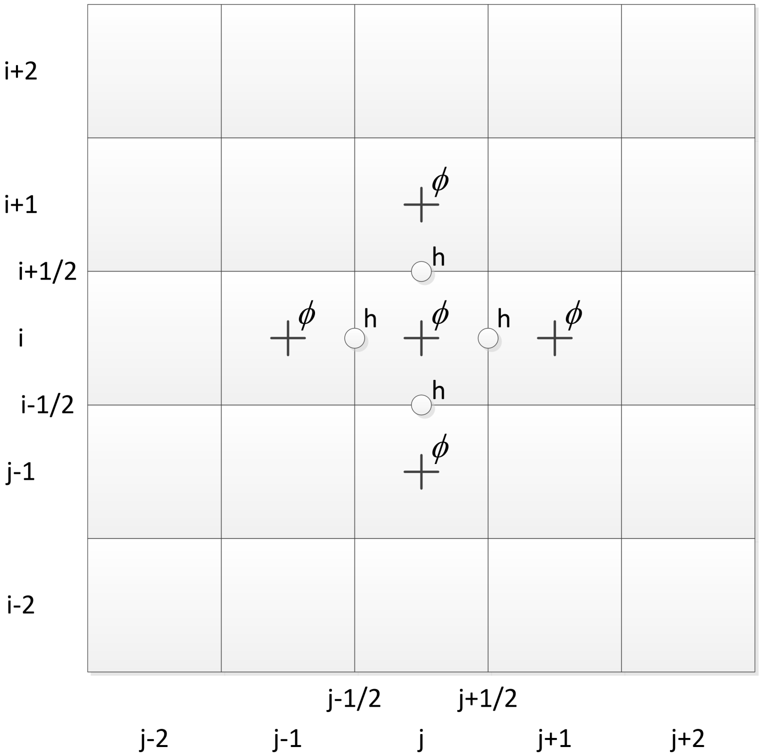

Fig. S8. Staggered-grid system for the finite difference method for Equation S6.

a)

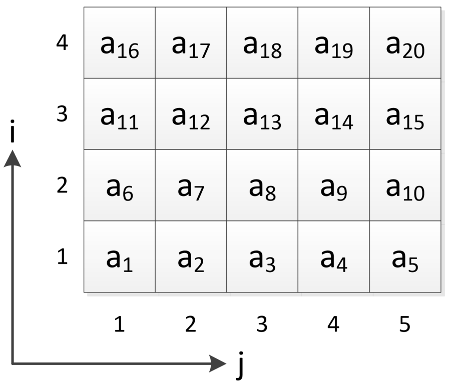

b)

I,j	1,1	1,2	1,3	1,4	1,5	2,1	2,2	2,3	2,4	2,5	3,1	3,2	3,3	3,4	3,5	4,1	4,2	4,3	4,4	4,5
1,1	a_1	a_2				a_6														
1,2	a_1	a_2	a_3				a_7													
1,3		a_2	a_3	a_4				a_8												
1,4			a_3	a_4	a_5				a_9											
1,5				a_4	a_5					a_{10}										
2,1	a_1					a_6	a_7				a_{11}									
2,2		a_2				a_6	a_7	a_8				a_{12}								
2,3			a_3				a_7	a_8	a_9				a_{13}							
2,4				a_4				a_8	a_9	a_{10}				a_{14}						
2,5					a_5				a_9	a_{10}					a_{15}					
3,1						a_6					a_{11}	a_{12}				a_{16}				
3,2							a_7				a_{11}	a_{12}	a_{13}				a_{17}			
3,3								a_8				a_{12}	a_{13}	a_{14}				a_{18}		
3,4									a_9				a_{13}	a_{14}	a_{15}				a_{19}	
3,5										a_{10}				a_{14}	a_{15}					a_{20}
4,1											a_{11}					a_{16}	a_{17}			
4,2												a_{12}				a_{16}	a_{17}	a_{18}		
4,3													a_{13}				a_{17}	a_{18}	a_{19}	
4,4														a_{14}				a_{18}	a_{19}	a_{20}
4,5															a_{15}				a_{19}	a_{20}

Fig. S9. Detail to construct the matrix $\vec{A}$ for Equation S10. a) Demonstation of matrix $\vec{A}$ with sizing of (4×5), b) Sparse matrix of the demonstation $\vec{A}$.
